# Activation of Type I and III Interferon Response by Mitochondrial and Peroxisomal MAVS and Inhibition by Hepatitis C Virus

**DOI:** 10.1371/journal.ppat.1005264

**Published:** 2015-11-20

**Authors:** Silke Bender, Antje Reuter, Florian Eberle, Evelyne Einhorn, Marco Binder, Ralf Bartenschlager

**Affiliations:** 1 Department of Infectious Diseases, Molecular Virology, Heidelberg University, Heidelberg, Germany; 2 Division of Virus-Associated Carcinogenesis, German Cancer Research Center (DKFZ), Heidelberg, Germany; 3 Master BioSciences, Département de Biologie, École Normale Supérieure de Lyon, Université de Lyon, Lyon, France; The University of Chicago, UNITED STATES

## Abstract

Sensing viruses by pattern recognition receptors (PRR) triggers the innate immune system of the host cell and activates immune signaling cascades such as the RIG-I/IRF3 pathway. Mitochondrial antiviral-signaling protein (MAVS, also known as IPS-1, Cardif, and VISA) is the crucial adaptor protein of this pathway localized on mitochondria, peroxisomes and mitochondria-associated membranes of the endoplasmic reticulum. Activation of MAVS leads to the production of type I and type III interferons (IFN) as well as IFN stimulated genes (ISGs). To refine the role of MAVS subcellular localization for the induction of type I and III IFN responses in hepatocytes and its counteraction by the hepatitis C virus (HCV), we generated various functional and genetic knock-out cell systems that were reconstituted to express mitochondrial (mito) or peroxisomal (pex) MAVS, exclusively. Upon infection with diverse RNA viruses we found that cells exclusively expressing pexMAVS mounted sustained expression of type I and III IFNs to levels comparable to cells exclusively expressing mitoMAVS. To determine whether viral counteraction of MAVS is affected by its subcellular localization we employed infection of cells with HCV, a major causative agent of chronic liver disease with a high propensity to establish persistence. This virus efficiently cleaves MAVS via a viral protease residing in its nonstructural protein 3 (NS3) and this strategy is thought to contribute to the high persistence of this virus. We found that both mito- and pexMAVS were efficiently cleaved by NS3 and this cleavage was required to suppress activation of the IFN response. Taken together, our findings indicate comparable activation of the IFN response by pex- and mitoMAVS in hepatocytes and efficient counteraction of both MAVS species by the HCV NS3 protease.

## Author Summary

Mammalian cells developed several defense mechanisms against viral infection. One major strategy involves pattern recognition receptors (PRRs) recognizing non-self motifs in viral RNA and triggering the production of type I and III interferon (IFN) that induce an antiviral state. One central signaling molecule in this cascade is MAVS (Mitochondrial Antiviral Signaling protein), residing on mitochondria, mitochondria-associated membranes of the endoplasmic reticulum, and peroxisomes. Here we characterized the role of mitochondrial and peroxisomal MAVS for the activation of the IFN response and their counteraction by the hepatitis C virus (HCV), a major causative agent of chronic liver disease with a high propensity to establish persistence. By using various functional and genetic knock-out cell systems reconstituted to express exclusively mitochondrial or peroxisomal MAVS, we observed comparable activation of type I and III IFN response by either MAVS species. In addition, we found that the HCV protease residing in nonstructural protein 3 (NS3) efficiently cleaves MAVS independent from its subcellular localization. This cleavage is required for suppression of the IFN response and might contribute to HCV persistence. Our results indicate a largely localization-independent activation of the IFN response by MAVS in hepatocytes and its efficient counteraction by the HCV NS3 protease.

## Introduction

Vertebrates developed several defense mechanisms against invading pathogens. Upon viral infection foreign RNA or DNA is introduced into the host cell where it is detected by highly conserved pattern recognition receptors (PRRs) sensing distinct non-self motifs [1–7]. Well known examples of PRRs are RIG-I (Retinoic acid-Induced Gene I) and MDA5 (Melanoma Differentiation-Associated protein 5) that both are cytosolic RNA helicases recognizing primarily 5’-triphosphorylated and long (i.e. >2,000 nucleotides) double stranded (ds) RNA, respectively [1, 3, 8]. Upon interaction with RNA, both RIG-I-like receptors (RLR) induce a signaling cascade which leads to the production of type I and III Interferon (IFN) as well as IFN stimulated genes (ISGs). In the case of RIG-I, RNA interaction induces conformational changes, rendering the CARD (Caspase Activation and Recruitment Domain) accessible for ubiquitination by TRIM25 and subsequent interaction with the signal adaptor protein MAVS (Mitochondrial Antiviral-Signaling protein; also known as IPS-1, Cardif or VISA) [9–13]. MAVS is a ubiquitously expressed protein consisting of an N-terminal CARD, a proline-rich region and a single transmembrane domain at the very C-terminus. Activation of MAVS induces a prion-like oligomerization of the protein, forming large signaling platforms [14]. These platforms lead to the activation of NFκB and IFN regulatory factor 3 and 7 (IRF3, IRF7), which upon nuclear translocation drive the expression of IFNs and other cytokine genes [9–12, 15]. In principle the same pathway is utilized upon activation of MDA5 [14, 16, 17].

The IFN system consists of three classes that are grouped according to the receptor to which they bind. Type II IFN is primarily produced by T cells and natural killer cells in response to the recognition of infected cells. Type I IFNs include IFN-β, which is encoded by a single gene and synthesized by most cell types, especially fibroblasts, and IFN-α, which is encoded by a gene cluster of 13 genes and predominantly synthesized by leukocytes. Type III IFN has only been described recently and includes three members: IFN-λ1, IFN-λ2 and IFN-λ3 that are also known as IL-29, IL-28A and IL-28B, respectively [18, 19]. Recently a new member of this group was discovered, designated IFN-λ4, which is a frameshift variant arising from a peculiar polymorphism in the IL28 gene locus that is predictive for the outcome of therapy of chronic hepatitis C [20]. IFN-λ is produced by several cell types including hepatocytes [21, 22] where it contributes to the control of hepatitis C virus infection (HCV) [23, 24] Moreover, type I and III IFNs use different receptors, with type III receptors being composed of two chains: the ligand binding chain IFNλR1 and the accessory IL-10R2 subunit that is shared with the receptor for cytokines of the IL10 family [24]. Tissue distribution of the type III IFN receptor is by and large restricted to epithelial cells and hepatocytes in humans [25, 26]. Although type I and type III IFN use different receptors, it is believed that they both signal via the JAK/STAT pathway to induce a very similar set of more than 300 ISGs [23, 27].

HCV is a member of the genus Hepacivirus within the family *Flaviviridae*. A hallmark of HCV is its high propensity to persist. In fact, around 80% of HCV infections become persistent and persistently infected individuals have a high risk to develop chronic liver disease, including liver cirrhosis and hepatocellular carcinoma [28, 29]. The HCV particle is enveloped and contains a single-strand RNA genome of positive polarity. This ~9,600 nucleotides long RNA encodes a polyprotein that is cleaved into 10 products. Processing of the polyprotein is mediated, in part, by a viral serine-type protease that forms the N-terminal domain of nonstructural protein (NS) 3. Activation of this protease requires the viral cofactor NS4A that intercalates into the protease domain and, in addition, anchors the NS3/4A complex to intracellular membranes [30]. Apart from polyprotein cleavage, NS3/4A was found to counteract the activation of the IFN response by efficient cleavage of MAVS at amino acid 508 [10]. Consistent with the localization of the NS3 protease active site, the cleavage site in MAVS resides close to the membrane surface, thus liberating MAVS from the membrane and blocking RLR signaling function, both *in vitro* and *in vivo* [10, 31–33]. While these studies focused on MAVS localizing to mitochondria and mitochondria-associated membranes (MAMs), a distinct membrane compartment that links this organelle to the endoplasmic reticulum [32], MAVS was also reported to localize to peroxisomes to induce a rapid type I IFN-independent ISG response [34]. Moreover, it was reported that mitochondrial (mito) MAVS induces IFN-β and IFN-λ whereas peroxisomal (pex) MAVS only triggers an IFN-λ response [35]. However, the relative contribution of mito- and pexMAVS to the induction of the antiviral state and counteraction of the MAVS variants by viral infection remains largely unexplored.

In this study we evaluated the relative contribution of mito and pexMAVS to the activation of the type I and III IFN response in hepatocytes upon infection with various RNA viruses. In addition, we determined the control of either MAVS species by HCV. We found that mito- and pexMAVS activated type I and III IFN response with comparable efficiency and kinetics and both MAVS variants were potently counteracted by HCV.

## Results

### Determination of the subcellular localization of endogenous MAVS and establishment of cell lines with organelle-targeted MAVS variants

**Fig 1:**
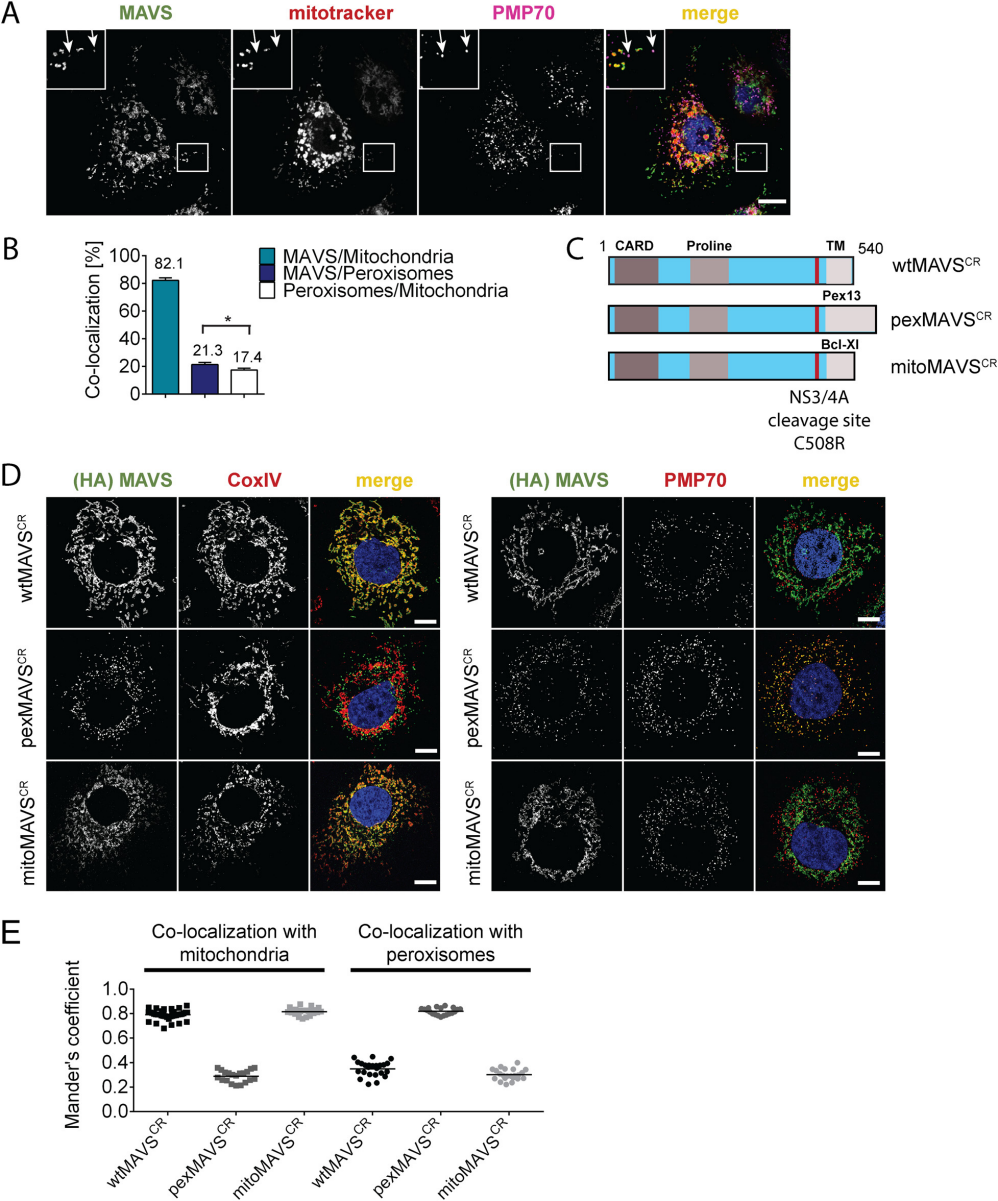
Subcellular localization of endogenous and engineered MAVS variants. (A) Subcellular localization of MAVS in Huh7 cells was determined by immunofluorescence. White arrows indicate co-localization of MAVS (green) with the peroxisomal marker PMP70 (purple). Mitochondria were stained with mitotracker (red) while nuclei were stained with DAPI. Scale bar, 10 μm. (B) Quantification of subcellular localization of MAVS on mitochondria and peroxisomes as determined with the Mander’s overlap coefficient. In addition, co-localization of peroxisomes with mitochondria was determined. Bars indicate the standard error. *, P≤0.05. (C) Organelle-targeted MAVS^CR^ (i.e. MAVS that is NS3/4A protease cleavage resistant) constructs were designed by replacing the transmembrane (TM)-domain (light grey) of wild type (wt) MAVS with either the TM-domain of *Pex13* (pexMAVS) or *Bcl-Xl* (mitoMAVS). The HCV NS3/4A cleavage site was mutated to obtain a cleavage resistant (CR) MAVS protein (C508R). The MAVS coding sequence is indicated in light blue and includes the CARD as well as the proline-rich sequence (grey boxes). (D) Co-localization of MAVS variants with given marker proteins (red) was determined in Huh7-NS3/4A expressing cells transduced with wt-, pex- or mitoMAVS^CR^-HA (green) by using immunofluorescence. Scale bar, 20 μm. (E) The degree of co-localization was quantified by calculating the Mander’s overlap coefficient. Each dot represents a single cell; at least 20 cells were analyzed per condition.

With the aim to characterize the activation of the interferon (IFN) response after viral infection by pex- and mitoMAVS we first determined the subcellular localization of endogenous MAVS on both organelles in the HCV-permissive human hepatoma cell line Huh7. Consistent with earlier reports endogenous MAVS primarily co-localized with mitochondria and, to a lesser extent, with peroxisomes as determined by co-staining with the marker protein PMP70 [9, 32, 34] (Fig 1A). Quantification of the MAVS distribution revealed that ~80% of the protein was localized on mitochondria and ~20% on peroxisomes (Fig 1B). However, ~17% of peroxisomes resided in close proximity to mitochondria, precluding the precise allocation of MAVS to either organelle. Thus, the total amount of pexMAVS might range between 3–20%. To study the impact of pex- and mitoMAVS separately, we utilized a strategy reported earlier [34] to generate MAVS variants exclusively localizing to either organelle. This was achieved by replacing the C-terminal transmembrane (TM) region of wild type MAVS (wtMAVS) with either the TM region of PEX13 (peroxisomal location; pexMAVS) or the TM region of Bcl-Xl (mitochondrial location; mitoMAVS). (Fig 1C). By using quantitative immunofluorescence, we confirmed the organelle-specific subcellular localization of these MAVS variants (Fig 1D).

To avoid competition between endogenous and ectopically expressed MAVS variants we attempted to reduce the levels of endogenous MAVS by using siRNA- and shRNA-mediated knock-down approaches; however, expression levels were at best reduced by 70%, which we considered to be insufficient for functional assays. To overcome this limitation we stably expressed the HCV NS3/4A protease, which is known to efficiently cleave MAVS off the mitochondria [10, 36], thus rendering MAVS inactive and interrupting signaling. Cleavage of MAVS occurs at the membrane-proximal cysteine residue 508, which we replaced by an arginine residue to render MAVS cleavage resistant [10, 37]. Variants containing this C508R point mutation are designated MAVS^CR^ throughout this report. Combining MAVS^CR^ with the organelle-specific TMs thus offered a possibility to study pex- and mitoMAVS in diverse human cell lines expressing the NS3/4A protease. To this end, we utilized Huh7 and A549 cells where MAVS localization to the respective organelle was confirmed by quantitative immunofluorescence analyses (Fig 1D and 1E and S1 Fig, respectively). In both cell lines we observed highly significant co-localization of pexMAVS^CR^ with peroxisomes and mitoMAVS^CR^ with mitochondria, while wtMAVS^CR^ mainly localized to mitochondria, with a small proportion localizing to peroxisomes, consistent with the subcellular distribution of endogenous MAVS (cf. Fig 1A and 1B).

Prior to conducting functional assays with these cell lines, we first determined whether wild type (i.e. C508) pexMAVS would be depleted from our cells by NS3/4A-mediated cleavage, because only then we would be able to measure IFN activation exclusively by exogenous organelle-specific MAVS. To allow measurement with high sensitivity we constructed a fusion protein composed of enhanced green fluorescent protein (eGFP) fused with a nuclear localization signal (NLS) and an organelle-specific targeting sequence (MAVS-C-terminal domain, MAVS-CTD) (S2A and S2B Fig). Analogous to an earlier report [38], upon cleavage of MAVS-eGFP the cleavage product is liberated from the membrane and translocated into the nucleus, which could be easily monitored by fluorescence imaging. Cells stably expressing NS3/4A or the non-functional protease mutant NS3/4A-S139A (S2C Fig) [39] were transfected with either one of the eGFP-NLS-MAVS-CTD constructs and subcellular eGFP distribution was determined. First, we confirmed localization of eGFP-NLS-tagged wt- and pexMAVS-CTD at peroxisomes in naïve Huh7 cells (S2B Fig). As expected, in cells expressing NS3/4A-S139A the eGFP distribution pattern of the MAVS variants was consistent with mitochondrial and peroxisomal localization (S2D Fig, left panel). In contrast, in NS3/4A-expressing cells transfected with eGFP-NLS-wtMAVS-CTD, the eGFP signal was exclusively detected in the nucleus reflecting highly efficient cleavage as reported earlier (S2D Fig, upper right panel) [10, 37, 38]. Importantly, analogous cleavage efficiency was also found with eGFP-NLS-pexMAVS-CTD (S2D Fig, lower right panel). These results demonstrate that HCV NS3/4A efficiently cleaves MAVS irrespective of its subcellular localization on mitochondria or peroxisomes.

### Comparable activation of the type I and III IFN system by expression of MAVS proteins localizing to peroxisomes or mitochondria

**Fig 2:**
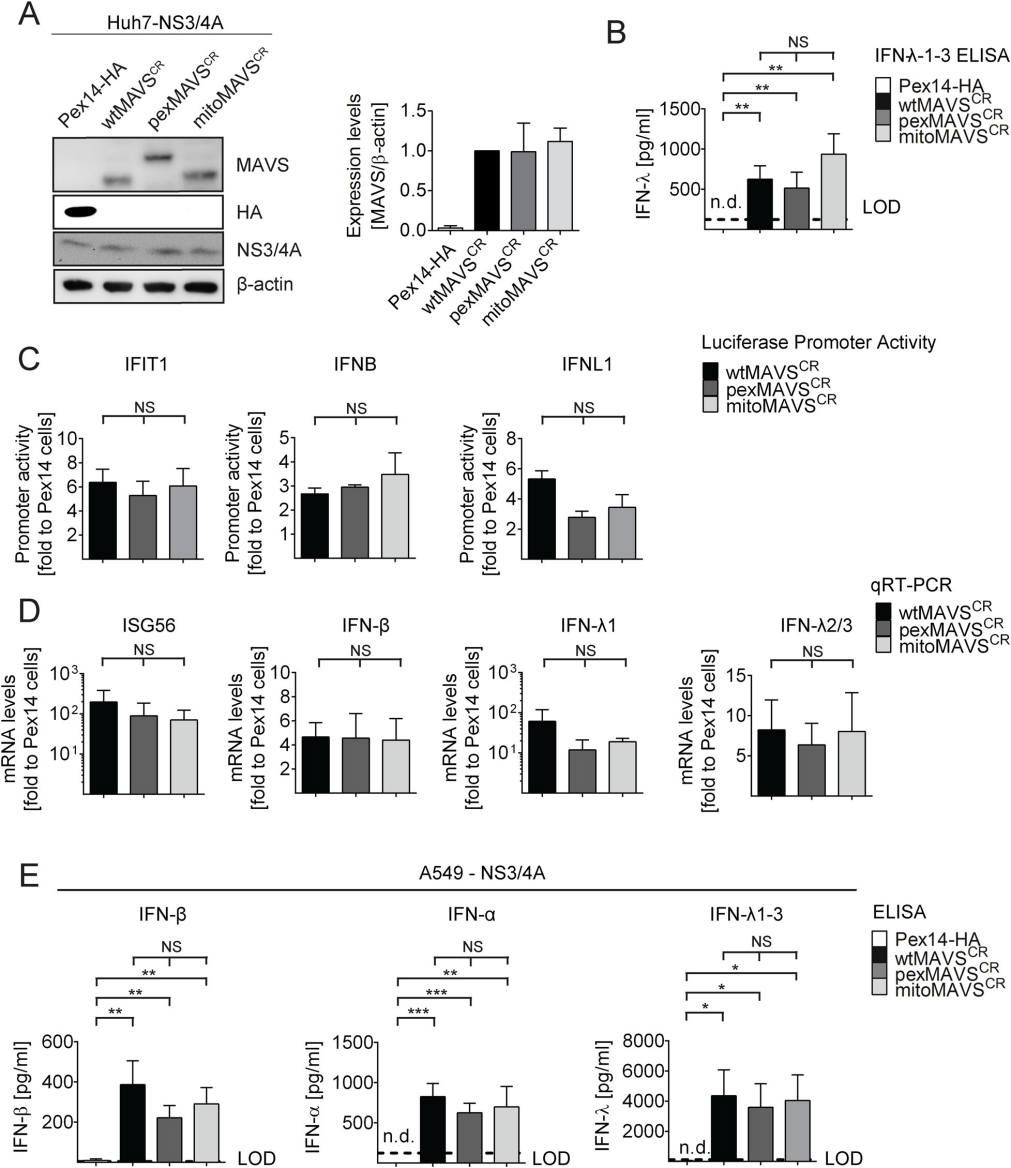
Comparable activation of the IFN system by MAVS proteins localizing to peroxisomes or mitochondria in Huh7 and A549 cells. (A) Huh7-NS3/4A expressing cells were transduced with non-cleavable wt-, pex- or mitoMAVS^CR^ or with Pex14-HA that served as control. Expression levels of MAVS^CR^ variants were determined by Western blot. The quantification in the right panel displays MAVS abundance, normalized to β-actin, relative to wtMAVS^CR^ expression levels (set to 1). Mean values from three independent experiments are shown. (B-D) Activation of the IFN response by overexpression of MAVS variants. (B) Huh7 cell culture supernatants were collected and IFN-λ1–3 protein levels were measured by ELISA. The dashed line indicates the limit of detection (LOD). N.d., not detectable. (C) For reporter-based analyses, firefly luciferase reporter plasmids were co-transfected with a SV40-based Renilla luciferase plasmid to normalize for transfection efficiency. Values for each reporter were normalized to those obtained for Pex14-HA transduced cells (set to 1). (D) Quantification of mRNA amounts of ISG56 and indicated IFNs as determined by qRT-PCR. Values were normalized to those obtained for Pex14-HA transduced cells (set to 1). All data were normalized to GAPDH using the ΔΔct method (E) A549 NS3/4A-expressing cells were transduced with lentiviruses containing non-cleavable wt-, pex- or mitoMAVS^CR^ as well as Pex14-HA that served as control. Cells were stimulated by overexpressing MAVS variants, cell culture supernatants were collected and IFN protein levels were measured by ELISA. The dashed line indicates the limit of detection (LOD). N.d., not detectable. Data represent the mean from four independent experiments. Bars indicate the standard error. *, P≤0.05; **, P≤0.005; ***, P≤0.0005; NS, not significant. N.d., not detectable.

Having confirmed that NS3/4A expressing cells are a valid model system for functional MAVS depletion and are suited to determine the capacity of organelle-specific MAVS^CR^ variants to induce the IFN response, we transduced NS3/4A overexpressing Huh7 cells with lentiviral vectors encoding organelle-specific MAVS^CR^ (Fig 2A). Consistent with earlier reports, we observed an intrinsic activation of the IFN response upon overexpression of MAVS as revealed by increased levels of IFN-λ in the supernatant of transduced cells [9, 40] (Fig 2B). This was not due to the lentiviral particles themselves since lentiviruses encoding the unrelated peroxisomal protein Pex14-HA did not induce IFN-λ expression. Apart from the activation of *IFNL*, we also observed an activation of IRF3 as determined by luciferase-based IFIT1, IFNB and IFNL1 promoter reporter assays (Fig 2C). Importantly, this activation was found with each MAVS variant and both, in the reporter assay and when measuring mRNA levels of endogenous ISG56 or various IFNs (Fig 2D). Moreover, analogous results were obtained with the IFN-competent cell line A549 (Fig 2E and S3 Fig) and with 293T cells (S4 Fig) demonstrating that the observed phenotypes are not specific to human hepatoma cells.

To exclude possible confounding effects exerted by NS3/4A in a MAVS cleaveage-independent manner [41, 42], we transiently transduced naïve A549 cells and A549-MAVS^KO^ cells with pex- or mitoMAVS variants. Of note, we observed a comparable induction of type I and type III IFN production, arguing that NS3/4A does not interfere with IFN activation, at least in this experimental setup (S5A and S5B Fig). We also determined the kinetics of IFN response activation by using microarrays to determine the transcriptome of wt-, mito- and pexMAVS-expressing cells. While most ISGs were upregulated to a similar degree, we observed subtle differences in induction kinetics for a few ISGs (S5C Fig). Although these differences were not consistent throughout the time course, it is possible that pex- and mitoMAVS have slightly different IFN induction capabilities.

### Comparable activation of type I and III IFN response by peroxisomal or mitochondrial MAVS upon virus infection

**Fig 3:**
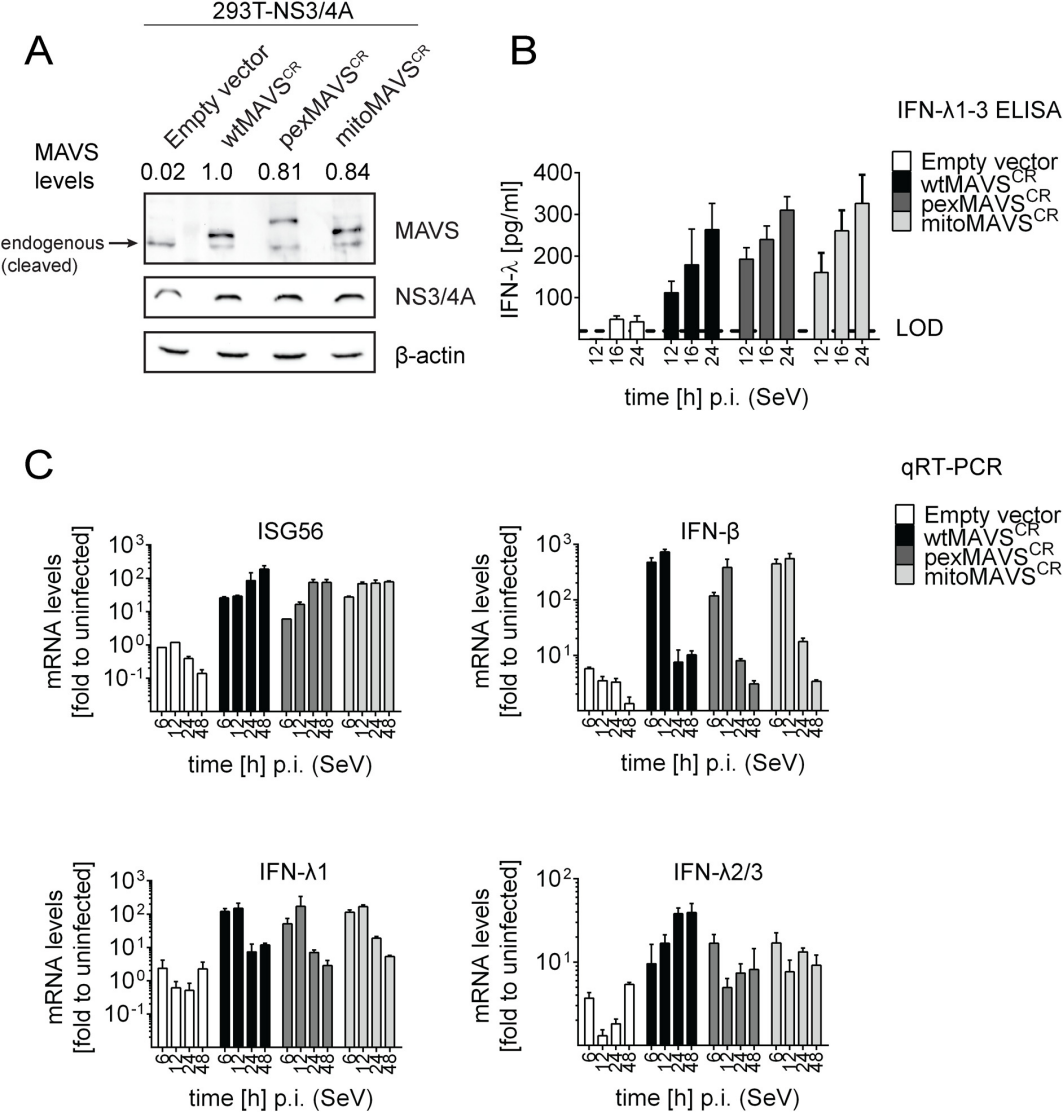
Comparable activation of type I and III IFN response by peroxisomal or mitochondrial MAVS upon virus infection. (A) Lysates of 293T cells stably expressing NS3/4A and various forms of non-cleavable MAVS^CR^ or an empty vector were analyzed by Western blot. Numbers above each lane refer to MAVS expression levels normalized to β-actin and wtMAVS^CR^ expression that was set to one. (B) Cells were infected with Sendai virus (SeV) with an MOI of 3. The amount of IFN-λ released at given time points into the cell culture supernatant was determined by ELISA. The dashed line represents the limit of detection (LOD). (C) Cells were infected with SeV and total RNA was extracted at time points specified in the bottom of each panel. mRNA amounts were quantified by qRT-PCR. All data were normalized to GAPDH using the ΔΔct method. A representative experiment of a total of three independent experiments is shown. Each value was measured in quadruplicates; mean values and standard errors are shown.

Having shown that expression of pexMAVS and mitoMAVS were able to induce type I and III IFN responses, we next compared these MAVS variants for their capacity to induce the IFN response upon viral infection. We reconstituted MAVS activity in 293T cells overexpressing HCV NS3/4A (293T-NS3/4A) by lentiviral transduction of MAVS^CR^ variants. As shown in Fig 3A, expression levels of the MAVS proteins in the cell pools were comparable, although wtMAVS abundance was ~20% higher as compared to the variants. Importantly, we only used cell pools that were not pre-activated by MAVS transduction, which we achieved by infecting the cells with MAVS-encoding lentiviruses at a very low MOI of 0.1 and passaging them under selective pressure. To investigate the IFN response we used the single stranded RNA virus Sendai Virus (SeV), which is specifically sensed by RIG-I [3, 7]. When we infected 293T-NS3/4A cells transduced with the empty vector with SeV or with other RNA viruses, we detected only a minor IFN response, which might be due to residual amounts of non-cleaved MAVS (0.2%) (Fig 3B, 3C and S6 Fig, respectively). However, in cells expressing mitochondrial or peroxisomal MAVS, IFN-λ expression was potently induced and amounts released into the culture supernatant of SeV-infected cells were well comparable between pexMAVS^CR^ and mitoMAVS^CR^ cells (Fig 3B). Consistent with this result we observed induction of endogenous ISG56, IFN-β and type III IFN expression upon SeV infection and these responses barely differed between the MAVS variants (Fig 3C). As reported earlier [43, 44], IFN-β expression was rapidly and very transiently induced and this also applied to IFN-λ1. In contrast, activation of IFN-λ2/3 expression was much more sustained and did not decline throughout the 48 h observation period. Activation of both type I and III IFN expression as well as ISG56 was also found when we infected the cells with other RNA viruses: Vesicular Stomatitis Virus (VSV) and Newcastle Disease Virus (NDV), which are both negative strand ssRNA viruses, and the dsRNA virus reovirus (S6 Fig). We note that kinetics and magnitudes of activation differed between these viruses, likely reflecting their diverse replication strategies. Nevertheless, comparable activation by mito- and pexMAVS was detected in all cases. Response levels were higher in case of wtMAVS, which might be due to the higher expression of this protein as compared to the MAVS variants (Fig 3A). Alternatively, highest IFN response might require both mito- and pexMAVS as suggested earlier [34]. Although we observed minor differences in ISG and IFN response in 293T cells between pex- and mitoMAVS we note that these differences were not consistent between different experiments. Taken together, we observed efficient MAVS-dependent activation of the type I and III IFN response upon virus infection, independent of the subcellular localization of MAVS.

### Activation of the interferon response by mitochondrial and peroxisomal MAVS in reconstituted MAVS^-/-^-mouse embryonic fibroblasts

**Fig 4:**
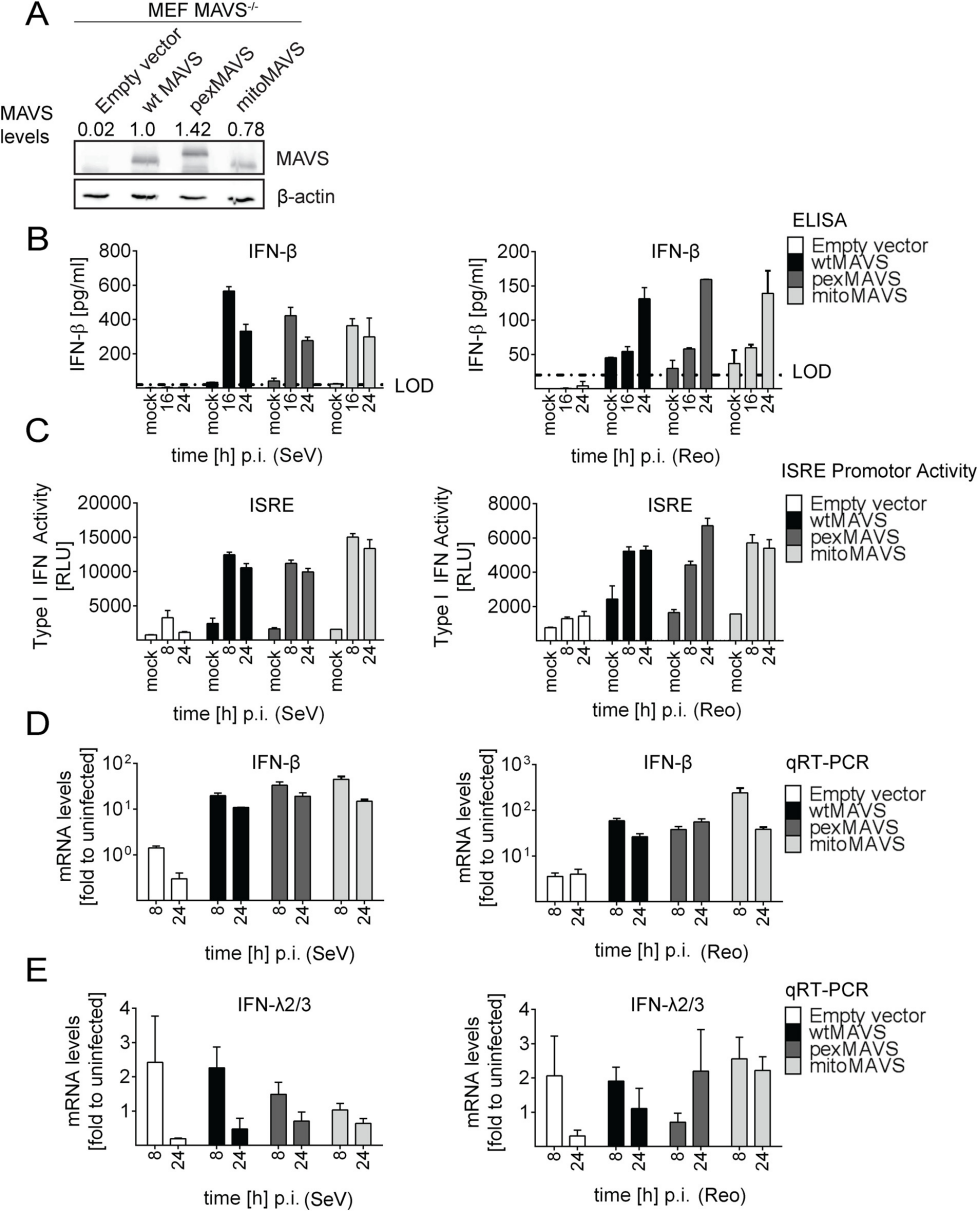
No difference in virus-induced activation of type I and III IFN response in MAVS^-/-^ mouse cells expressing peroxisomal and mitochondrial MAVS. (A) Mouse Embryonic Fibroblasts (MEFs) from MAVS^-/-^ knockout mice were transduced with lentiviral expression constructs encoding wt-, pex- or mitoMAVS or with the empty control vector. Expression of MAVS variants was validated by Western blot. Numbers above each lane refer to the expression level of the respective MAVS variant after normalization to β-actin and wtMAVS^CR^ that was set to one. (B, C) Amounts of IFN-β released from MAVS-reconstituted MEFs at different time points after virus infection into the culture supernatant and antiviral activity contained therein. (B) Cells were infected with SeV (MOI = 5) or Reo virus (MOI = 50) and IFN-β protein levels were quantified by ELISA. Dashed line represents the limit of detection (LOD). (C) Culture supernatants of MAVS-reconstituted MEFs harvested at different time points after SeV or Reo virus infection were inactivated by UV irradiation and used to inoculate L929 reporter cells. Eight hours later, cells were lysed and luciferase activity was determined. (D, E) Cells treated as described in panel (B) were harvested at given time points and amounts of IFN-β (D) or IFN-λ2/3 (E) mRNA were determined by qRT-PCR. All data were normalized to the housekeeping gene GAPDH and to the respective uninfected control (set to 1). Each figure in panels B-D shows a representative experiment of four independent repetitions, each conducted in technical triplicates. Mean values and standard errors are shown.

In the experiments described so far, we degraded endogenous MAVS from the mitochondria and peroxisomes by using the HCV NS3/4A protease. Under these conditions we were not able to detect an IFN response in these cells unless we reconstituted with MAVS^CR^ variants, demonstrating efficient blockade of MAVS-dependent signaling via mitochondria and peroxisomes. However, we could not exclude undesired effects exerted by either HCV NS3/4A overexpression or cleaved cytoplasmic MAVS. Therefore, we made use of mouse embryonic fibroblasts (MEFs) derived from a mouse line lacking endogenous MAVS (MAVS^-/-^) and reconstituted these cells by stable expression of wtMAVS, pexMAVS or mitoMAVS (Fig 4A). These cell pools were infected with SeV or reovirus, and analyzed for type I IFN production. At different time points after infection supernatants were harvested and secreted IFN-β was quantified by ELISA. While in MEFs lacking MAVS no IFN could be detected, in cells ectopically expressing organelle-specific MAVS similar levels of IFN-β were determined at 16 h post infection (Fig 4B). These results were further confirmed by the IFN reporter cell line L929-ISRE-Firefly-Luciferase, which is a reliable type I IFN bioassay as reported earlier [45]. Supernatants taken from virus-stimulated cells were UV-inactivated prior to adding to the reporter cell line. As expected, in MAVS^-/-^ MEFs without reconstituted MAVS no type I IFN was produced upon infection with SeV or reovirus (Fig 4C). However, consistent with our results obtained in NS3/4A-expressing cells, we detected comparable amounts of type I IFN in the supernatants of MEFs expressing wt-, pex- or mitoMAVS. Analogous results were obtained when we quantified the mRNA amounts of endogenous IFN-β in infected and functionally reconstituted MAVS^-/-^ MEFs (Fig 4D). A rapid and profound activation was observed in cells infected with either virus and no difference was detected between the MAVS variants. In contrast, induction of IFN-λ2/3 expression could not be detected in this cell system, arguing that the antiviral activity released into the culture supernatant was exclusively mediated by type I IFN (Fig 4E). In summary, these results corroborate the conclusion that mito and pexMAVS can induce the type I IFN response with comparable efficiency.

### Peroxisomes are dispensable as signaling platforms for type I and III IFN response

**Fig 5:**
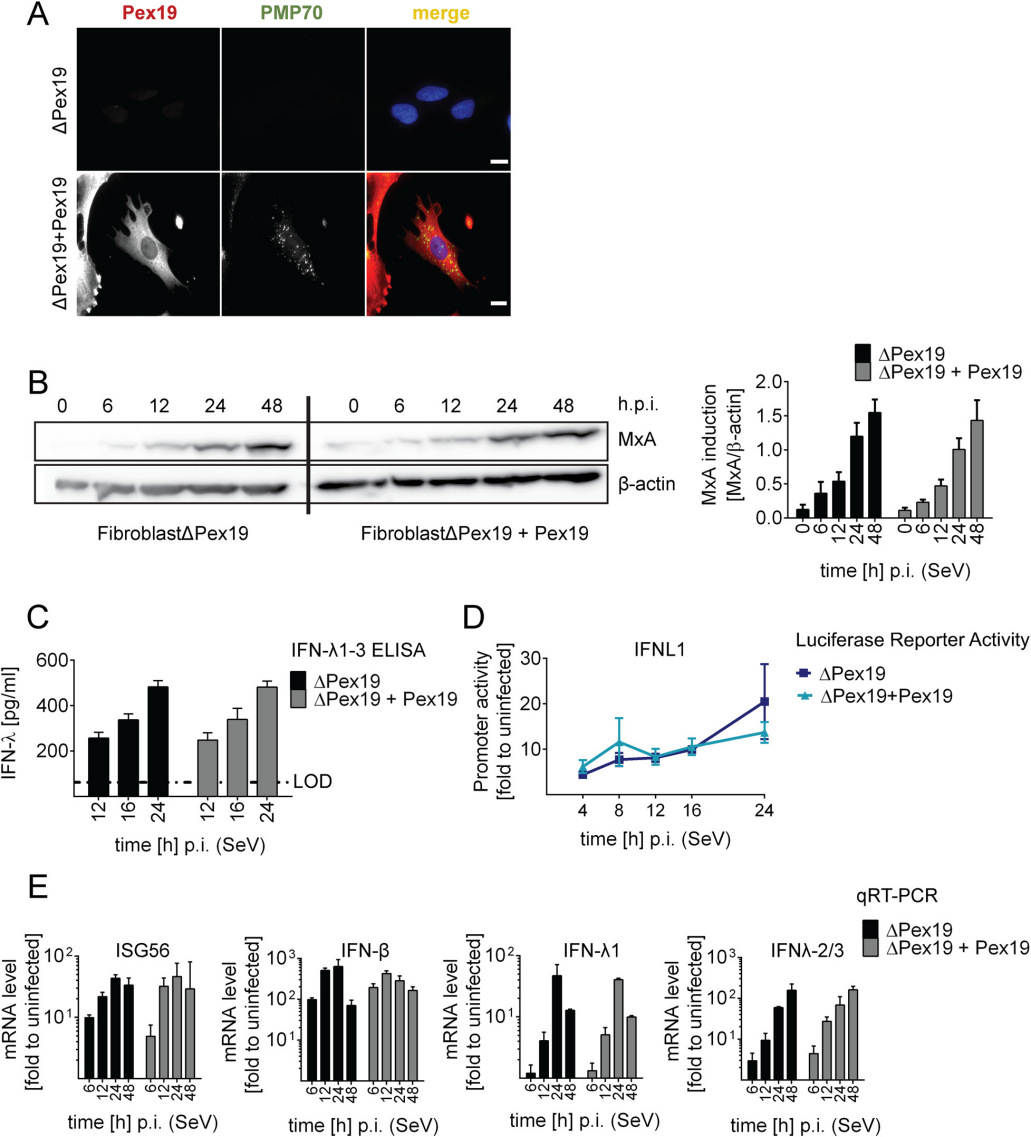
Activation of type I and III IFN response in peroxisome-deficient human cells. (A) Deficiency of peroxisome formation of the human fibroblast cell line ΔPex19 that does not contain peroxisomes was rescued by stable expression of Pex19 (ΔPex19+Pex19) as determined by immunofluorescence for Pex19 (red) and PMP70 (peroxisome marker, green). Nuclei were counterstained with DAPI. Scale bar, 40 μm. (B-D) Both cell lines were infected with SeV (MOI = 3) and samples were collected at indicated times points. (B) Total protein lysates of infected cells were analyzed for MxA abundance by Western blot. MxA-specific signals were quantified by using the Lab Image ID software package and values were normalized to β-actin that served as loading control. Values correspond to the mean of four independent experiments and standard errors. (C) Cell culture supernatants were harvested at given time points after SeV infection and amounts of IFN-λ1–3 were measured by ELISA. The dashed line indicates the limit of detection (LOD). (D) Fibroblasts were transfected with an IFN-λ-firefly luciferase reporter construct and 24 hours later infected with SeV. Cells were harvested at time points specified in the bottom and firefly luciferase activity was measured. Values were normalized to transfection efficiency as determined with a co-transfected SV40-based Renilla luciferase plasmid and are expressed relative to uninfected control cells (set to 1). (E) Total RNA was prepared from SeV-infected cells at time points specified in the bottom of each panel and amounts of mRNAs given on the top of each panel were determined by qRT-PCR. All data were normalized to GAPDH and uninfected control cells (set to 1). Mean values of a representative experiment conducted with technical triplicates are shown. Bars indicate the standard error. All experiments were performed at least three times.

The results described so far were based on the use of engineered MAVS variants with defined subcellular localization. Since we could not exclude that a small fraction of MAVS, undetectable by our assays, might have been mislocalized, we took advantage of cells lacking peroxisomes. By definition, these cells are unable to mount MAVS signaling platforms at peroxisomes, thus offering an alternative approach to study the activation of the IFN response in the absence of pexMAVS. This is the case with the patient-derived human fibroblast cell line ΔPex19 [46, 47], which naturally lacks the peroxisomal factor Pex19 required for budding of peroxisomes from the ER membrane [48]. These cells do not have peroxisomes but the peroxisomes can be restored by stably expressing recombinant Pex19 [47]. Indeed, upon ectopic expression of Pex19, we could detect peroxisomes, as revealed by staining with the peroxisomal marker PMP70 (cell line ΔPex19+Pex19), which were absent in non-reconstituted cells (Fig 5A).

To determine activation of the type I and III IFN response in the absence or presence of peroxisomes we infected the two cell lines with SeV and monitored the kinetics of MxA protein accumulation that is induced by type I and III IFN. As shown in Fig 5B, activation of this ISG did not require peroxisomes and induction kinetics were virtually identical. Moreover, SeV infection also induced robust type III IFN production as measured by ELISA in culture supernatants and by a luciferase reporter assay based on the IFNL1 promoter (Fig 5C and 5D). Consistently, mRNA levels of ISG56 were induced in both cell lines already 6 h post infection and increased over time (Fig 5E). Importantly, both type III and type I IFN expression was induced with IFN-β showing a rapid response kinetic whereas activation of IFN-λ1 and IFN-λ2/3 was slower.

### HCV triggers an IFN-λ response in Huh7 MAVS^KO^ cells rescued with cleavage-resistant mitochondrial and peroxisomal MAVS

**Fig 6:**
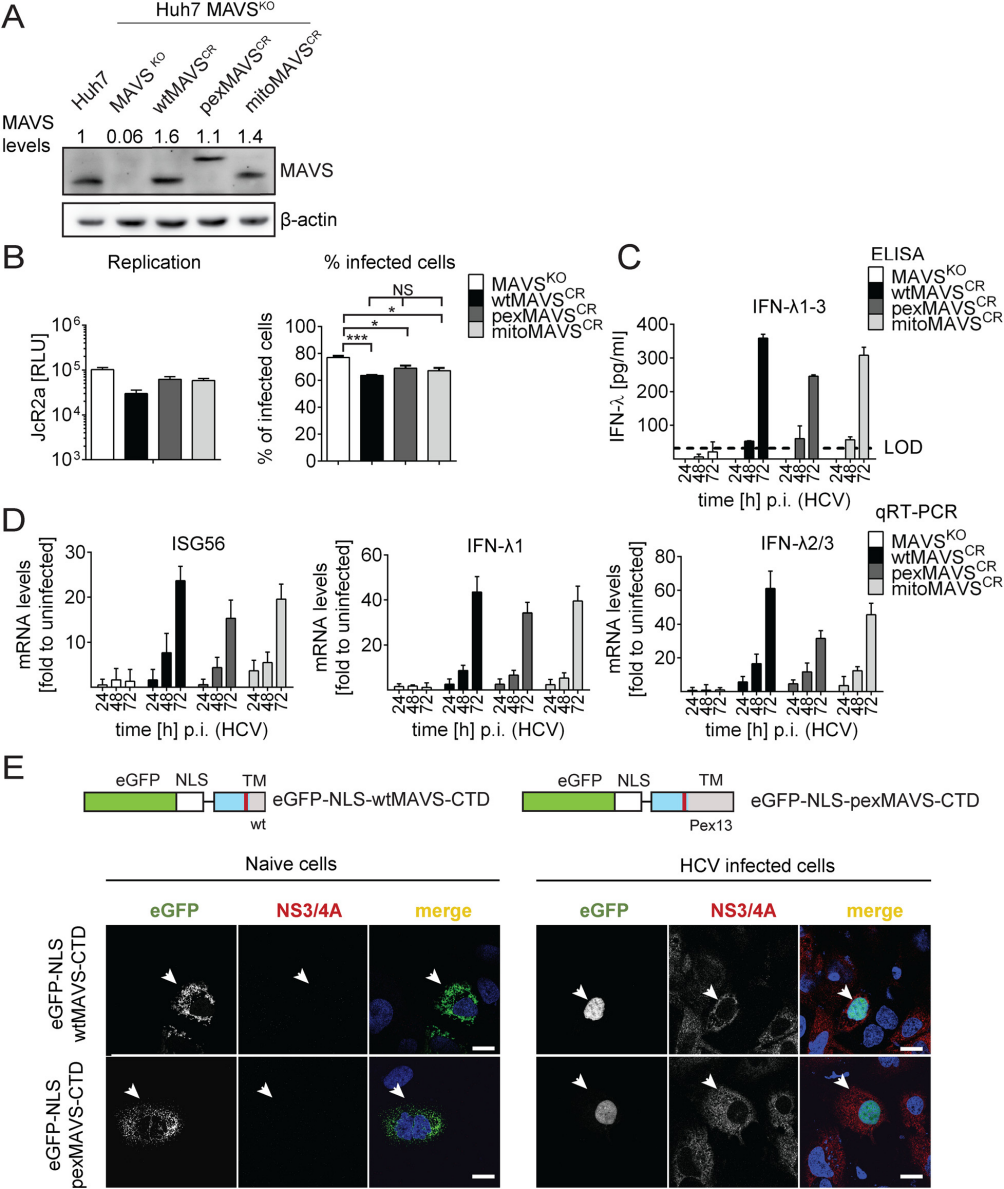
HCV activates type III IFN response via peroxisomal and mitochondrial MAVS to a comparable extent. (A) Huh7 MAVS^KO^ cell lines were generated using the CRISPR/Cas system. Cleavage resistant wt-, pex- and mitoMAVS were expressed in these cells by lentiviral transduction. Expression levels of the proteins were quantified by Western blot. Numbers above each lane refer to abundance of a given MAVS variant, normalized to β-actin and endogenous MAVS that was set to one. (B) Cell lines were infected with either the HCV reporter virus JcR2a (MOI = 1) or Jc1 (MOI = 1). Seventy two hours later HCV replication in JcR2a-infected cells was determined by luciferase assay whereas Jc1-infected cells were analyzed by NS5A-specific immunofluorescence. The percentage of infected cells was determined by counting at least 500 cells for each condition. (C) Cells were infected with the HCV isolate Jc1 (MOI = 5) and amounts of IFN-λ1–3 released into cell culture supernatants were determined by ELISA. The dashed line represents the limit of detection limit (LOD). N.d., not detectable. (D) Total RNA of infected cells was extracted and mRNA amounts of ISG56 and type III IFNs were determined by qRT-PCR. All data were normalized to GAPDH using the ΔΔct method and are expressed relative to uninfected control cells (set to 1). A representative experiment performed with technical triplicates is shown. Bars indicate the standard error. All experiments were conducted three times and with two additional Huh7 knockout cell clones. (E) Huh7 cells were infected with Jc1 (MOI = 5) and transfected 40 hours later with expression constructs encoding GFP-tagged CTD of wt- or pexMAVS (for construct design see Fig 2A). Note that HCV-mediated cleavage of the reporter results in nuclear accumulation of the eGFP signal. White arrow indicates an HCV infected cell. Scale bar, 20 μm.

Having established the comparable activation of type I and III IFN response by mito- and pexMAVS, we wanted to clarify the relative impact of either MAVS species on the IFN response during infection with HCV. To this end we generated Huh7 MAVS knockout cell lines (Huh7 MAVS^KO^) by targeting the first exon of the coding sequence of MAVS with the CRISPR/Cas9 system [49]. Since in only a fraction of cells knock-out is achieved, we generated three independent knock-out cell clones, whereby each clone was generated by using each time the same sgRNA. For each of these cell clones cell pools were established expressing NS3/4A protease cleavage resistant MAVS^CR^ variants localizing to the mitochondria or peroxisomes as well as wild type MAVS^CR^ (Fig 6A; results obtained for only one cell clone are shown). In the initial set of experiments we determined HCV permissiveness of the cell lines by infecting them with an HCV reporter virus encoding *Renilla* luciferase (JcR2a). In all tested cell pools we observed robust HCV replication as determined by luciferase assay (Fig 6B, left panel) and analysis of cells by immunofluorescence staining revealed an infection efficiency of 60–80% (Fig 6B, right panel, and S7 Fig). Interestingly, after introducing the MAVS^CR^ variants, the relative number of infected cells showed a minimal, yet statistically significant reduction, but there was no difference between organelle-specific MAVS variants (Fig 6B, right panel). Consistent with our earlier report [50], upon HCV and SeV infection there was no type I IFN production detectable in these Huh7 cells, (S8 Fig). In contrast, a robust type III IFN response was observed after infection with HCV. This response was first detected 48 h after infection (~55 pg/ml IFN-λ) and increased massively in the subsequent 24 h reaching ~250 pg/ml with pexMAVS^CR^ cells and 305 pg/ml with mitoMAVS^CR^ cells (Fig 6C). These quantitative differences corresponded well to the variations of expression levels of the MAVS variants (cf. Fig 6A). Consistently, mRNA levels of endogenous ISG56, IFN-λ1 and IFN-λ2/3 increased 48 h after HCV infection and reached maximum levels 72 h after infection (Fig 6D). These results were not a cell clone specific effect, because well comparable results were obtained with (a) two independent MAVS^KO^ cell clones, each reconstituted with MAVS^CR^ variants and analyzed in parallel and (b) Huh7 cells stably expressing NS3/4A to purge endogenous MAVS from intracellular membranes and reconstituted with MAVS^CR^ variants (S9 Fig).

### Efficient control of peroxisomal MAVS in hepatitis C virus infected cells

With the aim to characterize control of pexMAVS in HCV infected cells, we utilized the eGFP-NLS-MAVS-CTD fusion proteins described above (cf. S2A Fig), which were expressed in Huh7 cells 36 h after HCV infection by transient transfection. As expected, in uninfected cells we observed predominantly mitochondrial localization of the wtMAVS marker protein (Fig 6E, left panel). However, upon HCV infection cleaved wtMAVS-eGFP translocated into the nucleus, demonstrating efficient cleavage (Fig 6E, right panel). Importantly, pexMAVS was also cleaved quantitatively in HCV-infected Huh7 cells as revealed by the exclusive nuclear localization of the marker protein.

**Fig 7:**
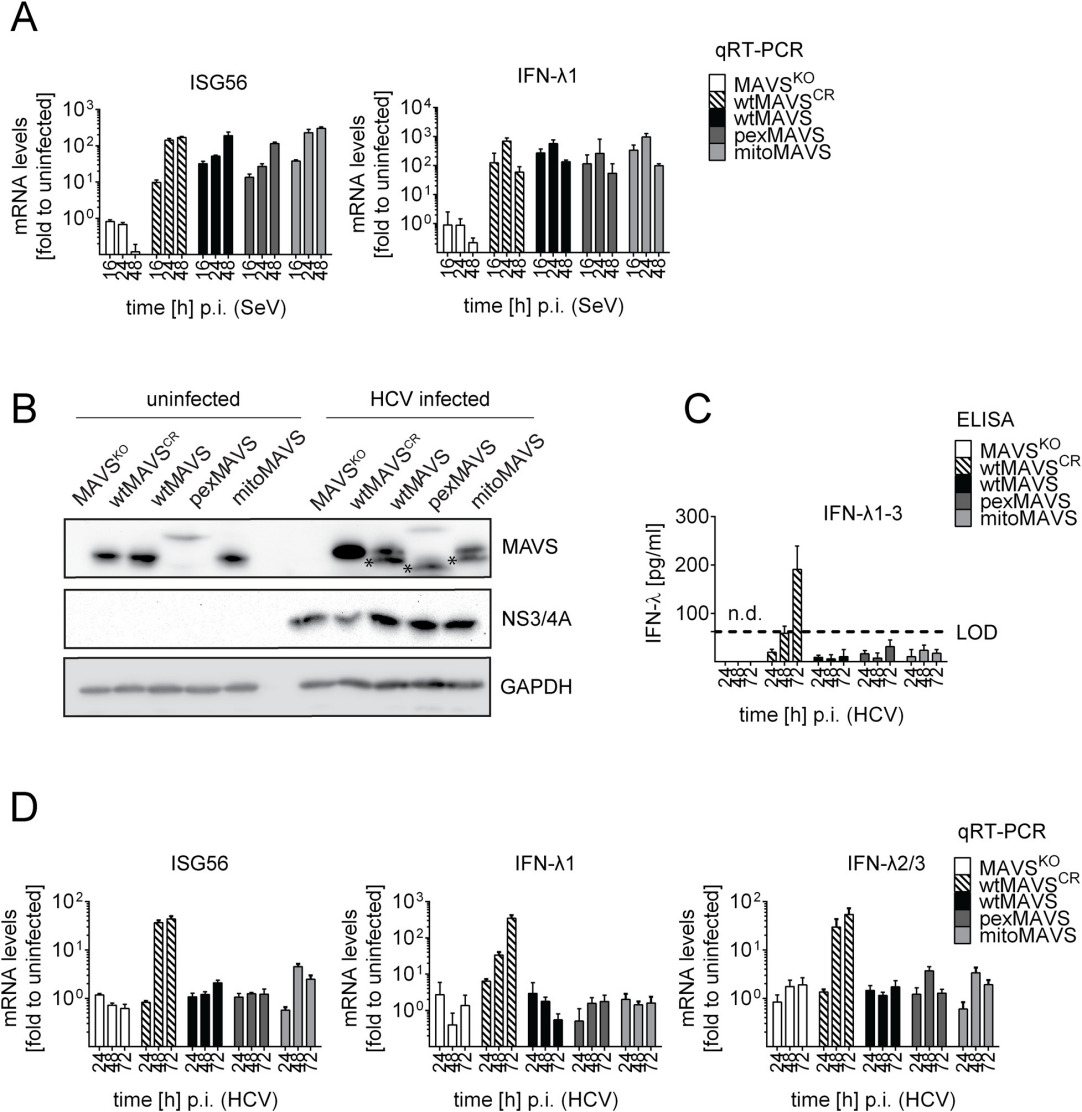
Blunting of the interferon response upon HCV infection occurs independent from MAVS subcellular localization. Huh7-MAVS^KO^ cells were rescued with cleavable wt-, pex- or mitoMAVS or cleavage-resistant wtMAVS^CR^. (A) Cells were infected with SeV (MOI = 5) for periods specified in the bottom of each panel. Total RNA was isolated and amounts of ISG56 and IFN-λ1 mRNAs were determined by qRT-PCR. All data were normalized to GAPDH and uninfected control cells (set to 1). (B) Cells were infected with HCV and 48 hours later MAVS cleavage and HCV infection was determined by Western blot using antibodies specified in the right of each panel. Asterisks indicate cleavage products of MAVS variants. (C) Cell culture supernatants were harvested at given time points after HCV infection and amounts of IFN-λ1–3 were measured by ELISA. The dashed line indicates the limit of detection (LOD). (D) Amounts of ISG56 and IFN-λs mRNA were determined by qRT-PCR. Values were normalized to those obtained for uninfected cells. All data were normalized to GAPDH using the ΔΔct method.

Taking advantage of this cell system, we next determined the impact of MAVS cleavage and MAVS subcellular localization on activation of the IFN response by HCV in an infection-based system. This was possible because the used cells were highly permissive for HCV, mounted a robust IFN response, but did not suppress HCV replication, thus excluding confounding effects on activation kinetics as a result of impaired virus replication. To this end we constructed Huh7 MAVS^KO^ cells expressing exclusively HCV cleavable, i.e. wild type full-length MAVS variants localizing specifically to peroxisomes or mitochondria. All cell lines responded comparably to SeV infection by upregulation of ISG56 and IFN-λ1, thus validating their functionality (Fig 7A). Of note, the ISG response induced by SeV and Reo virus was only detectable at late time points after infection (> 4 hours; S10 Fig). As expected, upon HCV infection all MAVS variants were efficiently cleaved, yet also non-cleaved MAVS was detected by Western blot (Fig 7B). Since uncleaved MAVS might be produced by non-infected cells, we utilized an immunofluorescence-based single cell readout. We found that in HCV-infected cells MAVS was not detectable and this was independent from the subcellular localization of MAVS (S11 Fig). Consistent with this efficient MAVS cleavage in HCV-infected cells was the almost complete block of IFN-λ production and ISG56 response (Fig 7C and 7D). This inhibition was specific and not observed in cells reconstituted with the cleavage-resistant MAVS (wtMAVS^CR^).

Taken together we demonstrate that both mitochondrially and peroxisomally localized MAVS induce a robust type III IFN response in Huh7 cells with comparable efficiency. Importantly, both MAVS variants are efficiently controlled in HCV-infected cells by the NS3/4A protease, thus blocking IFN response irrespective of MAVS localization.

## Discussion

Activation of the IFN response by the RLRs RIG-I and MDA5 critically depends on MAVS that relays the signal via several protein kinases to NFκB and IRF3, which in turn promote the transcription of IFN-β, IFN-λ and several ISGs [9–12]. MAVS was originally found to localize to mitochondria, but more recent studies also described MAVS on MAMs and peroxisomes [9, 32, 34]. Although in this study we could confirm peroxisomal localization of MAVS in the human hepatoma cell line Huh7, it is still unclear how MAVS is targeted to this organelle. While the transmembrane region in the C-terminal part of MAVS appears to be responsible for targeting to mitochondria [9] and peroxisomes [34], the molecular details remain to be clarified. Three possibilities can be envisaged how MAVS is targeted to peroxisomes. First, regarding mitochondria, the majority of peroxisomal proteins is translated by free polyribosomes in the cytosol [51–53] and they are post-translationally incorporated into newly formed peroxisomes via Pex19p [53]. Second, MAVS is present on ER membranes [32]. Since peroxisomes are derived from the ER, MAVS might be incorporated into new peroxisomal membranes by chance when they bud off [54]. Third, MAVS might shuttle from mitochondria to peroxisomes via a specialized transport system called mitochondrial-derived vesicles as described for other proteins [55]. It remains to be determined by which route MAVS is targeted to peroxisomes or whether several of these routes are used. In any case, we observed efficient cleavage of pexMAVS by the HCV protease. This might occur at the ER, prior to MAVS “loading” onto peroxisomes. NS3/4A might also be recruited to peroxisomes where cleavage could occur. Consistent with the latter assumption it has been reported that a fraction of NS3/4A co-localizes with peroxisomes [32].

We and others observed that overexpression of MAVS *per se* was sufficient to activate the RLR pathway [9, 40]. However, there was no consistent difference between organelle-specific MAVS variants and this we observed with several cell lines and by using direct (ELISA, qRT-PCR and Western blotting) and indirect detection methods (luciferase-based promoter assays). Moreover, after RLR stimulation by viral infection, comparable responses were induced by pexMAVS or mitoMAVS. Of note, efficient antiviral signaling was also detected in the human fibroblast cell line ΔPex19, which lacks peroxisomes altogether, and no difference in activation kinetics or magnitude was found in this cell line after functional reconstitution of peroxisomes.

It has been reported that peroxisomal proteins, in the absence of peroxisomes, either stay in the cytosol, are degraded or mistargeted to other organelles such as mitochondria [56–59]. Consistently, immunofluorescence staining of MAVS in ΔPex19 cells revealed a typical mitochondrial distribution and the abundance of MAVS in cells with or without peroxisomes was well comparable. Therefore, we assume that mitochondrial MAVS might compensate for peroxisomal MAVS in cells lacking peroxisomes. In any case, the activation of type I and III IFN in ΔPex19 cells shows that peroxisomes are dispensable for induction of this antiviral response. To corroborate this assumption we attempted stable knock-down of Pex19 in Huh7 cells, but this was not possible because of cytotoxicity. Moreover, transient knock-down was limited because peroxisomes not only renew from the ER, but also renew from already existing peroxisomes (G. Dodt, Thübingen University, personal communication), thus requiring extended periods of Pex19 depletion which could not be achieved by transient knock-down.

Although we did not observe a difference in activation of the IFN response by mito- or pexMAVS in various cell lines and experimental approaches, Dixit and colleagues described a rapid, type I IFN-independent signaling of pexMAVS in MAVS^-/-^ MEFs after reovirus infection [34]. Furthermore, the same group recently reported that upon viral infection pexMAVS induces IFN-λ1, but not IFN-β in Huh7 cells after knockdown of endogenous MAVS expression [35]. Consistent with this data, we could confirm IFN-λ1 activation by pexMAVS in MAVS knock-out Huh7 cells as well as IFN-λ2/3 production as determined by ELISA, luciferase reporter assay and qRT-PCR analysis. Moreover, for each condition and approach we detected comparable induction of type III IFNs by wt-, pex- and mitoMAVS. However, we did observe an induction of type I IFN by peroxisomally localized MAVS in i) MAVS^-/-^ MEFs after stimulation with reovirus or SeV, ii) Huh7 and A549 cells after ectopic expression of MAVS variants and iii) 293T cells with a functional (NS3/4A-mediated) block of endogenous MAVS and expressing organelle-specific cleavage-resistant MAVS variants. These results are at odds with the earlier studies that concluded a selective activation of *IFNL* by pexMAVS. Currently, we can only speculate about possible reasons for the discrepancy. One possibility is the use of different experimental set ups, most notably the MAVS constructs. Common to the present and the earlier studies is the replacement of the C-terminal TM domain of MAVS by pex- and mito-targeting sequences (Pex13 and Bcl-XI, respectively; Fig 1C). However, while we fused MAVS with the targeting sequence at amino acid position 514 of MAVS, the MAVS fusion constructs employed in the earlier studies retained MAVS only up to amino acid residue 500, which might affect signaling capacity of the MAVS fusion proteins. Indeed, activation of the IFN response by pex- and mitoMAVS truncated at position 500 seems rather low as compared to wtMAVS [34, 35]. An alternative explanation for the discrepant results reported here and in the two earlier studies might be differences in sensitivities of used read-out systems to measure type I IFN. In fact, we were unable to detect IFN-β in Huh7 cells by ELISA, but could detect low endogenous IFN-β activation by qRT-PCR and by promoter assay. This appears to be a specific property of Huh7 cells, because in several other cell lines we could detect robust IFN-β by pex and mitoMAVS.

Currently, we cannot exclude that a very small fraction of pexMAVS is localized on mitochondria. To address this possibility we attempted to separate peroxisomes, MAMs and mitochondria biochemically, but consistent with an earlier report [32] we found that subcellular fractionation was not sufficient for that purpose. However, we did find that gradual knockdown of mitoMAVS to a level still detectable by fluorescence microscopy profoundly reduced the ISG response after SeV infection (S12 Fig). Given that pex- and mitoMAVS efficiently induced the IFN response to comparable levels, the degree of activation observed with pexMAVS cannot be ascribed to small amounts of pexMAVS mislocalized to mitochondria and not detectable by fluorescence microscopy. Thus we conclude that the activation observed with pexMAVS is indeed mediated by MAVS residing at peroxisomes. We also note that mitoMAVS might not only localize on mitochondria, but as reported earlier, Bcl-Xl also localizes to the ER [60–62]. However, ER or MAM localization of mitoMAVS would not affect our conclusions.

By using Huh7-MAVS^KO^ cells expressing organelle-specific MAVS^CR^ variants, we observed a profound activation and secretion of type III IFN upon HCV infection (Fig 6). Surprisingly, while this IFN is biologically active, HCV replication in infected cells was barely affected. This might be due on one hand to impaired responsiveness of Huh7-MAVS^KO^ cells to IFN (S13 Fig), on the other hand to the limited IFN response of HCV in Huh7 cells [63]. Although this precludes studying the impact of IFN released from HCV-infected cells on viral replication and spread, it offers the possibility to characterize activation of the IFN response without confounding effects caused by a reduction in replication resulting from the antiviral state induced by IFN.

It has been proposed that HCV might induce type III IFN via non-cleaved pexMAVS [64]. However, we demonstrate that pexMAVS is efficiently cleaved in HCV-infected cells. While this result verifies the reported co-localization of NS3/4A with peroxisomes [32], we cannot exclude that a fraction of MAVS remains uncleaved. At the single cell level, despite Western Blot analysis indicating incomplete cleavage, MAVS was virtually non-detectable in HCV-infected cells. Thus, the apparent incomplete MAVS cleavage as detected by Western blot probably reflects incomplete infection of the used Huh7 cell cultures with uncleaved MAVS being produced by non-infected cells. The analogous might apply to MAVS cleavage as it was observed in the liver of HCV-infected patients [33]. Thus, the type III IFN response observed by our group and others [65–68] in primary human hepatocytes after HCV infection might result from a small fraction of non-cleaved MAVS or induction of other innate signaling pathways such as TLR3, rather than reflecting a specific role of peroxisomal MAVS.

The functional and biological differences of type I and III IFN remain poorly understood. Both classes of IFNs are produced after activation of RIG-I, MDA5 and TLR3 and both induce a very similar spectrum of ISGs via the JAK/STAT signaling pathway [69, 70]. However, the two IFN classes are produced in different tissues and bind to different receptors, IFNAR and IL28R, respectively, [18, 19] both of which also have distinct tissue distribution. Restriction of the IFN-λ receptor complex to epithelial cells and hepatocytes indicates a high importance of IFN-λ for control of infections with viruses of distinct tropism such as influenza or hepatitis viruses, respectively [65, 66, 71, 72]. Furthermore, stimulation of cells with IFN-β or IFN-λ1–3 induces a long-lasting ISG response, while IFN-α induces a much faster and less sustained ISG response [69]. Consistent with these reports we found that cells expressing only IFN-β and IFN-λ mount a similar ISG56 response, which was comparably induced by mitoMAVS and pexMAVS.

The observation that cleavage of MAVS by the NS3/4A protease completely blocks activation of the IFN response suggests that MAVS, which is not tethered to the membrane, loses its signaling capacity. Although purified MAVS protein forms prion-like structures even in solution *in vitro* and therefore independent of its transmembrane domain [15], we and others never detected an IFN response by cleaved MAVS inside cells. This might be due to the lower effective concentration of cleaved MAVS in the cytoplasm as compared to the *in vitro* conditions and to a rapid degradation of MAVS that has a half-life of only ~4 hours in HCV replicon-containing cells [9, 37].

In summary, our data indicate that peroxisomes appear to be dispensable in terms of signaling platforms in the type I and III IFN activation pathway, arguing for a redundant role of pex- and mitoMAVS. We observed comparable induction of type I and type III IFN by peroxisomal and mitochondrial MAVS upon viral infection and show that both, mitochondrial as well as peroxisomal MAVS are efficiently cleaved and inactivated in HCV-infected cells. Thus, HCV has evolved strategies to counteract this important IFN signaling molecule independent from its subcellular localization.

## Materials and Methods

### Cell culture

All cell lines were cultured in Dulbecco’s modified Eagle medium (DMEM, Life Technologies, Germany) supplemented with 10% fetal calf serum (GE Healthcare, Germany), 100 μg/ml penicillin, 100 μg/ml streptomycin (Sigma Aldrich, Germany), non-essential amino acids (Life Technologies, Germany) and maintained in a humidified incubator with 5% CO_2_ at 37°C. Stable cell lines were created by lentiviral transduction as described below and cultured in selection medium in the presence of 1 mg/ml G418 (Life Technologies, Germany), 5 μg/ml puromycin (Sigma Aldrich, Germany) and/or 5 μg/ml blasticidin (MP Biomedicals, USA). MAVS^-/-^ mouse embryonic fibroblasts, human fibroblasts deficient for Pex19 (ΔPex19) and mouse L929-ISRE-Firefly-Luciferase cells were kindly provided by Ulrich Kalinke (Hannover, Germany), Gabrielle Dodt (Tübingen, Germany) and Steeve Boulant, (Heidelberg, Germany), respectively.

### Virus production

Jc1 wild type HCV and the Renilla Luciferase reporter virus JcR2a were generated as described recently by using the plasmids pFKJFH1/J6/C_846_ΔG and pFKJFH1/J6/C_846_-JcR2a, respectively [73, 74]. Briefly, in vitro transcripts were purified by phenol/chloroform extraction and precipitated at room temperature using isopropanol. Ten microgram RNA was transfected by electroporation into 3.5x10^6^ Huh7.5 cells using the GenePulser system (Bio-Rad, Germany). Cell culture supernatant was harvested 24, 48 and 72 hours post electroporation, pooled, filtered through a 0.45 μm SteriCap (Millipore, Germany) and precipitated with polyethylene glycol (PEG) 800 (Applichem, Germany) in PBS for 72 hours at 4°C. After centrifugation for two hours at 8,000x g, the pellet was resuspended in DMEM and the titer was determined by limiting dilution assay using the TCID_50_ method (http://www.klinikum.uni-heidelberg.de/Downloads.126386.0.html). Vesicular Stomatitis Virus (a kind gift from Gert Zimmer, Mittelhäusern, Switzerland) was produced by infecting DF1 chicken embryo fibroblasts (MOI = 0.0001) and harvesting culture supernatant 48 hours later. Virus stocks were titrated by standard plaque assay. Sendai virus (kindly provided by Rainer Zawatzki, Heidelberg, Germany) and New Castle Disease Virus (a kind gift from Georg Kochs, Freiburg, Germany) were propagated in LSL Valo SPF embryonic chicken eggs (Lohmann Tierzucht, Germany). Shortly, eggs were incubated at 37.8°C with 60% humidity and turned every 24 hours. Eggs were infected at embryonic development day 12 with 10^3^ plaque forming units diluted in OptiMEM (Life Technologies, Germany). Allantoic fluid was harvested from 48 to 72 hours post infection, cellular debris was removed by centrifugation and the virus titer was determined by using the TCID_50_ method. Reo virus was kindly provided by Steeve Boulant (Heidelberg, Germany).

### Antibodies

The mouse monoclonal antibody 9E10 detecting NS5A domain III of HCV was kindly provided by Charles Rice (New York, USA). The Pex19-specific monoclonal antibody was a kind gift of Gabrielle Dodt (Tübingen, Germany). The mouse monoclonal NS3/4A antibody recognizing the NS3 helicase (2E3) was generated in cooperation with Hengli Tang (San Diego, USA). The mouse anti-MxA antibody was purchased from Georg Kochs (Freiburg, Germany). Other commercially available antibodies used in this study were mouse monoclonal antibodies recognizing β-actin (Sigma Aldrich, Germany), GAPDH (Santa Cruz Biotechnology, USA) and the HA-tag (Sigma Aldrich, Germany) as well as rabbit polyclonal antibodies recognizing MAVS (Enzo Life science, Switzerland), PMP70 (Abcam, United Kingdom) and CoxIV (Cell Signaling, Netherlands). Goat-anti-rabbit-Alexa-488, goat-anti-mouse-Alexa-488, donkey-anti-mouse-Alexa-568, goat-anti-rabbit-Alexa-568, chicken-anti-rabbit-Alexa-647 (all from Life Technologies, Germany) were used as secondary antibodies. For Western blot analysis anti-mouse and anti-rabbit antibodies, each coupled with horseradish peroxidase were used (Sigma, Germany).

### Plasmids and DNA cloning

Unless otherwise stated, plasmids were taken from the Orfeome cDNA library collection (Life Technologies, Germany) that is based on the pENTR221 vector. Organelle-specific targeting of MAVS was achieved by replacing the wild-type-transmembrane (TM) region of MAVS with the TM region of either Bcl-Xl (clone BC019307; aa 203–233) [75] or Pex13 (isolated from cDNA derived from Huh7 cells; aa 136–233) [76]) by using PCR-based mutagenesis. Cleavage resistant MAVS containing the C508R substitution was generated by QuickChange site directed mutagenesis (Agilent Technologies, Germany) as recommended by the manufacturer. To construct the organelle-specific MAVS-eGFP-NLS reporter, the C-terminal domain (CTD) of pex- and wtMAVS were inserted into the pCDNA6.2 vector (Life Technologies, Germany). All the other constructs were generated by inserting the respective gene into the pDONR207 vector and shuttling of the insert into the pWPI lentiviral transduction vector by using the Gateway cloning technology (Life Technologies, Germany) according to the instruction of the manufacturer. The type III IFN reporter pGL3-IFNλ1-Firefly-Lucifease was generated as previously described [77].

### Lentivirus production and generation of stable cell lines

Stable cell lines were generated as described earlier [78]. In brief, 1.2x10^6^ 293T cells were seeded into a 6 cm-diameter dish one day before transfection. Plasmids pMD.G (encoding the VSV G glycoprotein), pCMVΔR8.91 (encoding HIV gag-pol) and pWPI (encoding the gene of interest and a selection marker) were transfected in a 1:3:3 ratio into the cells by using the CaPhos Mammalian Transfection kit (Clontech Laboratories, France) according the manufacturer´s protocol. Supernatant was harvested 36, 48 and 72 hours post transfection and passed through a 0.45 μm filter. Target cells were seeded at the density of 8x10^4^ cells per well of a six-well plate and 24 hours later inoculated with different amounts of supernatant containing lentiviral particles The selection was started 36 hours post transduction. For MAVS transduction experiments lentiviral particles were purified by sedimentation onto a 20% sucrose cushion using a SW28 Ti rotor (Beckman Coulter, Germany) and two hours centrifugation at 20,000 rpm.

### Luciferase based reporter systems

RIG-I signaling in Huh7, 293T and A549 cells with organelle-specific MAVS was measured indirectly by using dual luciferase reporter constructs as previously described [79]. Briefly, cells were seeded into 96 well plates 24 hours before co-transfection with either IFIT1, IFNB, or IFNL1 promotor constructs pGL3B or p125-Firefly-Luciferase (kindly provided by Ganes Sen, Cleveland and Takashi Fujita, Tokyo, respectively [79, 80]). The co-transfected Renilla-Luciferase reporter plasmid pRL-SV40 (Promega, Germany) served as transfection control. Plasmid transfection was conducted with the Effecten transfection reagent (Qiagen, Germany) according to the instructions of the manufacturer. Cells were infected with different viruses 24 hours post transfection. For some experiments specified in the results section, cells were transfected with reporter plasmids 24 hours after lentiviral transduction and lysed 16 hours later. For mouse type I IFN bioassays, MEFs expressing organelle-specific MAVS variants were infected with either Sendai or Reo virus. Supernatant was harvested at given time points and stored at -80°C. Mouse L929-ISRE-Firefly-luciferase reporter cells [45] were seeded 24 hours prior to treatment. Reporter cells were stimulated with UV-inactivated supernatant and eight hours later firefly luciferase activity contained in cell lysates was determined.

### Quantitative RT-PCR

Total cellular RNA was isolated from single wells of a 24 well plate using the NucleoSpin RNA extraction kit (Macherey-Nagel, Germany) according to the manufacturer´s protocol. RNA analysis was carried out using the two-step qRT-PCR approach. Total RNA was reverse transcribed into cDNA and DNA amplicons were measured with SYBR Green (Bio-Rad, Germany). The following primers were used: human GAPDH: forward 5’-GAAGGTGAAGGTCGGAGTC-3’, reverse 5’- GAAGATGGTGATGGGATTTC-3’; human IFN-β: forward 5’- CGCCGCATTGACCATCTA-3’, reverse 5’-GACATTAGCCAGGAGGTTCTC-3’; human IFN-λ1: forward 5’-GCAGGTTCAAATCTCTGTCACC-3’, reverse 5’-AAGACAGGAGAGCTGCAACTC-3’; human IFN-λ2/3 forward 5’-CAGCTGCAGGTGAGGGA-3’, reverse 5’-GCGGTGGCCTCCAGAACCTT-3’; human ISG56: forward 5’-GAAGCAGGCAATCACAGAAA-3’, reverse 5’-TGAAACCGACCATAGTGGAA-3’; mouse GAPDH: forward 5’-AGGTCGGTGTGAACGGATTTG-3’, reverse 5’-TGTAGACCATGTAGTTGAGGTCA-3’; mouse IFN-β: forward 5’-CAGCTCCAAGAAAGGACGAAC-3’, reverse 5’-GGCAGTGTAACTCTTCTGCAT-3’; mouse IFN-λ2/3: forward 5’-AGGGTGCCATCGAGAAGAG-3’, reverse 5’-GTGGTCAGGGCTGAGTCATT-3’. Data were analyzed by using the ΔΔct method as described recently [81].

### Enzyme-linked immunosorbent assay (ELISA)

IFN-α pan (Mabtech, Sweden), IFN-β as part of VeriPlex Human Cytokine 16-Plex ELISA Kit (PBL-Interferon source, USA), IFN-λ1–3 (PBL-Interferon source, USA) and mouse IFN-β (BioLegend, USA) contained in the supernatant of cells were quantified according to the instructions of the manufacturers.

### Immunofluorescence

Cells were seeded on cover slips in a 24-well plate. In case of mitotracker staining (Life technologies, Germany), the dye was added 20 minutes before fixation with 4% paraformaldehyde (PFA) for 20 minutes. Cells were washed with PBS and permeabilized using 0.5% Triton X-100 for 5 minutes. After blocking of non-specific binding with 3% bovine serum albumin (BSA) in PBS for at least 20 minutes, cells were incubated with primary antibodies in 1% BSA for 60 minutes. After washing with PBS, cells were stained with secondary antibodies in 1% BSA. Nuclear DNA was stained with 4',6-diamidino-2-phenylindole (DAPI, MoBiTec, Germany). Cells were mounted on glass slides using Fluoromount-G (SouthernBiotech, USA). Pictures were taken on either a Leica SP2 confocal laser scanning microscope (Leica, Germany) or a Keyence BZ 9000 Imager (Keyence, Germany). Image processing including cropping, automatic contrast, merge, cell counting and Mander’s coefficient analysis were performed using the Fiji software package [82].

### Creating human knockout cell line

Knock-out of MAVS in Huh7 cells was achieved by using the CRISPR/Cas9 system as previously described [49]. In brief, three different single-guide RNAs (sgRNAs) were designed targeting the first and second exon of the coding region of MAVS with the help of the open source tool [83] and inserted into the lentiviral vector lentiCRISPR v2 (Addgene #52961) also encoding the Cas9 nuclease. The following sgRNAs were used: 5’ TCAGCCCTCTGACCTCCAGCG 3’ (#1), 5’ CGCTGGAGGTCAGAGGGCTGG 3’ (#2), 5’ AGGGGCTGCAGAGGGTAAACG 3’ (#3). Lentiviruses were produced as described above and 8x10^4^ cells were transduced three times using undiluted stocks of lentiviral particles encoding sgRNA #1, 2 or 3. All shown data were obtained by using a cell clone treated with sgRNA #1, but analogous results were obtained with cell clones generated with the two other sgRNAs. To establish MAVS knock-out cells, clonal selection was performed using single cell dilution in a 96-well plate. Knock-out was confirmed by Western blotting, immunofluorescence and functional tests.

### Small interfering RNA (siRNA)-mediated knockdown

Cells were seeded with a density of 3x10^4^ cells per well of a 24-well plate one day prior to transfection. Transfection was performed with Lipofectamine RNAiMax (Life technologies, Germany) according to the instruction of the manufacturer using a siRNA targeting MAVS (5’ CCCACAGGGUCAGUUGUAU 3’) and a control siRNA. Forty-eight hours post transfection cells were infected with Sendai virus. Knockdown efficiency was determined by Western blot analysis.

### Western blot analysis

Cells were lysed in 2x Laemmli buffer (200 mM Tris-HCl [pH8.8], 5 mM EDTA, 0.1% (w/v) Bromophenol Blue, 10% (w/v) Sucrose, 3% (w/v) SDS, 2% β-Mercaptoethanol) and heated to 98°C for 10 minutes. Proteins were separated by SDS-PAGE and blotted onto a PVDF membrane by either semi-dry or wet-blot (Bio-Rad, Germany). Membranes were blocked with 5% milk in PBS containing 0.1% Tween 20 for one hour at room temperature or overnight at 4°C. Membranes were incubated with primary antibodies overnight at 4°C or for 90 minutes at room temperature. Secondary antibodies tagged with horseradish peroxidase were incubated for one hour at room temperature. After washing with PBS containing 0.1% Tween 20, proteins were visualized by using the Western Lightning Plus-ECL reagent (PerkinElmer, USA) and an INTAS Advanced Fluorescence and ECL imager (INTAS, Germany) or a Curix 60 Developer device (AGFA, Germany). The Lab Image 1D software package (Kapelan BioImaging Solutions, Germany) was used for quantification of specific signals that were normalized to β-actin or GAPDH or wild type MAVS expression as specified in the results section.

### Microarray

Total RNA was purified as described above and analyzed using the Illumina HumanHT-12 v4 Expression BeadChip technology (Illuminia, USA) according to the protocols of the manufacturer. Data were processed using the software package R (http://www.r-project.org).

### Statistics

Statistical analysis of obtained data was performed using the GraphPad Prism software package (GraphPad software, USA). Unpaired t-tests were performed as described in the results section. Asterisks indicate ***, P-value ≤0.0005; **, P-value ≤0.005; *, P-value ≤0.05; ns, non-significant.

## Supporting Information

S1 Fig Subcellular localization of MAVS variants in A549 cells. (A) A549 cells stably expressing HCV NS3/4A were transduced with lentiviral vectors encoding for wt-, pex- or mitoMAVS^CR^-HA (green). Cells were analyzed by immunofluorescence using antibodies specified in the top. Nuclear DNA was stained with DAPI. Scale bar, 20 μm. (B) The degree of co-localization was quantified using Mander’s overlap coefficient; each dot represents a single cell. Twenty cells were analyzed per MAVS variant.
(TIF)

S2 Fig Cleavage of peroxisomal MAVS by the HCV NS3/4A protease. (A) Schematic representation of MAVS reporter constructs. The fusion proteins are composed of eGFP containing a nuclear localization signal (NLS), the C-terminal region (CT) of MAVS (light blue) spanning the NS3/4A protease cleavage site (red vertical line) and the wt or pex transmembrane (TM) domain for subcellular targeting. (B) Huh7 cells were transfected with eGFP-NLS-wtMAVS-CTD or eGFP-NLS-pexMAVS-CTD constructs. Co-localization of the reporter proteins with mitochondria (mitotracker, red) and peroxisomes (PMP70, purple) was determined by immunofluorescence. Scale bar, 10 μm. (C) Expression of the catalytically inactive HCV protease NS3/4A-S139A and the parental NS3/4A in stably transduced Huh7 cells was confirmed by Western blot; β-actin served as loading control. Empty control refers to cells transduced with the empty vector. (D) Huh7 cells expressing the inactive mutant (NS3/4A-S139A; left panel) or the parental protease (right panel) were transfected with expression constructs specified on the left and 24 hours later cells were fixed and analyzed by fluorescence microscopy. Nuclei were stained with DAPI. Scale bar, 20 μm.
(TIF)

S3 Fig No difference in the activation of the IFN response by peroxisomal and mitochondrial MAVS in a type I IFN competent cell line. (A) A549-NS3/4A expressing cells were transduced with lentiviruses containing non-cleavable wt-, pex- or mitoMAVS^CR^ as well as Pex14-HA that served as control. Cells were lysed 36 hours after transduction and expression of MAVS^CR^, endogenous cleaved MAVS and MxA were determined by Western blot using antibodies specified in the right of each panel. The relative expression levels of MAVS normalized to GAPDH and wtMAVS^CR^ (set to 1) are shown in the right panel (mean values of three independent experiments and standard errors). (B-C) Cells were stimulated by overexpressing MAVS variants. (B) To determine activation of the IFIT1, IFNB and *IFNL1* promoter (left, middle and right panel, respectively), cells were transfected with firefly luciferase reporter plasmids and a SV40-based Renilla luciferase plasmid to normalize for transfection efficiency. After 24 hours cells were lysed and luciferase activity was measured. (C) qRT-PCR analysis was performed to determine mRNA levels for the genes specified in the top of each panel. Values were normalized to GAPDH by using the ΔΔct method. Data represent the mean from four independent experiments. Bars indicate the standard error. *, P≤0.05; **, P≤0.005; ***, P≤0.0005; NS, not significant.
(TIF)

S4 Fig Transient overexpression of MAVS variants in 293T cells and activation of type I and III IFN response. (A) 293T cells stably expressing NS3/4A were transduced with lentiviral vectors encoding for non-cleavable wt-, pex- or mitoMAVS^CR^ as well as Pex14-HA that served as negative control. Expression of transduced genes was determined by Western blot. The quantification in the right panel displays MAVS expression levels normalized to β-actin and wtMAVS^CR^ that was set to one. Mean values and standard error of three independent experiments are given. (B) Thirty six hours after transduction cell culture supernatants were collected to measure IFN-λ1–3 protein levels by ELISA. Dashed line represents the limit of detection (LOD). N.d., not detectable. (C) To measure promoter activation firefly luciferase reporter plasmids specified on the top of each panel were co-transfected with a SV40-based Renilla luciferase plasmid. Values were normalized to those obtained for Pex14-HA (set to 1). (D) Amounts of mRNAs specified in the top of each panel were quantified by qRT-PCR. All data were normalized to the housekeeping gene GAPDH using the ΔΔct method. Bars indicate the standard error. All experiments were performed at least three times independently. *, P≤0.05; **, P≤0.005; ***, P≤0.0005; NS, not significant.
(TIF)

S5 Fig Transient overexpression of MAVS variants induces comparable levels of type I and III IFN as well as ISGs in A549 cells. (A) A549 and (B) A549-MAVS^KO^ cells generated by a CRISPR/Cas9 approach were transduced with lentiviral vectors encoding for wt-, pex- or mitoMAVS^CR^ as well as Pex14-HA that served as negative control and was used for normalization. Amounts of mRNAs specified in the top of each panel were quantified by qRT-PCR. All data were normalized to the housekeeping gene GAPDH using the ΔΔct method. Bars indicate the standard error. All experiments were performed at least three times independently. NS, not significant. (C) A549-MAVS^KO^ cells were transduced with lentiviral vectors encoding wt-, pex- or mito-MAVS and Pex14-HA as control. Total RNA was isolated 24, 48 and 72 hours post transduction (p.t.) and analyzed using whole-genome microarrays. Colors indicate the fold increase relative to Pex14-HA transduced cells. Every condition was performed in three independent experiments.
(TIF)

S6 Fig Comparable activation kinetics of type I and III IFN promoters by mitochrondrial and peroxisomal MAVS in virus-infected 293T cells. 293T cells stably expressing NS3/4A and non-cleavable wt-, pex- or mitoMAVS^CR^ or an empty vector were infected with VSV (MOI = 3), Reo virus (MOI = 50) or NDV (MOI = 5). Twenty four hours prior to infection cells were transfected with firefly luciferase reporter plasmids specific for (A) IFIT1, (B) IFNB or (C) IFNL1. A SV40-based Renilla-luciferase plasmid was co-transfected as control to normalize for transfection efficiency. Values for each reporter were normalized to uninfected control cells that were set to one. Each panel displays a representative experiment, each conducted with technical quadruplicates. Shown are the means and standard errors (not visible in all graphs because of low variation). All experiments were conducted three times.
(TIF)

S7 Fig Permissiveness of MAVS^KO^ Huh7 cells expressing NS3/4A protease cleavage-resistant MAVS variants. A Huh7 cell line with a CRISPR/Cas-mediated knockout of MAVS was generated and transduced with lentiviral vectors encoding cleavage resistant wt-, pex- or mitoMAVS^CR^. Cells were infected with the HCV isolate Jc1 (MOI = 1) and 48 hours later fixed and processed for immunofluorescence using antibodies recognizing MAVS (red) and NS5A (green). Scale bar, 100 μm. Note that the sporadic signals obtained with MAVS^KO^ cells are background frequently localizing in-between the cells.
(TIF)

S8 Fig Poor IFN-β induction in virus-infected MAVS^KO^ Huh7 cells reconstituted with MAVS variants. (A) Huh7 MAVS^KO^ cell lines expressing cleavage resistant wt-, pex- and mitoMAVS were infected with the HCV isolate Jc1 (MOI = 5). Total RNA was extracted and mRNA amounts of IFN-β were determined by qRT-PCR. All data were normalized to GAPDH using the ΔΔct method and are expressed relative to uninfected control cells (set to 1). (B) Analogous to panel (A), but cells were infected with SeV (MOI = 5). For each panel, a representative experiment performed with technical triplicates is shown. Bars indicate the standard error. All experiments were conducted three times and with two additional Huh7 knockout cell clones.
(TIF)

S9 Fig Activation of the interferon response in MAVS-reconstituted Huh7 cells after infection with HCV. (A) Huh7 cells stably expressing NS3/4A and non-cleavable wt-, pex- or mitoMAVS^CR^ or an empty vector were lysed and expression levels of endogenous MAVS and MAVS variants was determined by Western blot. Note the quantitative cleavage of endogenous MAVS by the HCV protease. β-actin served as loading control. Numbers above each lane refer to abundance of a given MAVS variant, normalized to β-actin and wtMAVS^CR^ that was set to one. (B) Cells were infected with the HCV reporter virus JcR2a (MOI = 1) and Luciferase activity was measured to monitor replication 72 hours post infection. (C) Huh7-NS3/4A-MAVS^CR^ cells were infected with the HCV isolate Jc1 (MOI = 5). Culture supernatants were harvested at time points specified in the bottom of the graph and amount of IFN-λ1–3 was determined by ELISA. The dashed line represents the limit of detection (LOD). N.d., not detectable. (D) HCV-infected cells were harvested at given time points, total RNA was extracted and mRNA levels of ISG56, IFN-λ1 and IFN-λ2/3 were determined by qRT-PCR. All data were normalized to GAPDH using the ΔΔct method and to uninfected control cells (set to 1). Shown are the means of a representative experiment, each time point measured in triplicates. Bars indicate the standard error. All experiments were done three times.
(TIF)

S10 Fig Kinetics of ISG56 activation in MAVS reconstituted Huh7-MAVS^KO^ cells. Huh7-MAVS^KO^ cells were rescued with cleavable wt-, pex- or mitoMAVS or cleavage-resistant wtMAVS^CR^. (A) Cells were infected with SeV (MOI = 5) or (B) Reo virus (MOI = 50) and harvested at different time points after infection that are specified in the bottom of each panel. Total RNA was isolated and amounts of ISG56 mRNA were determined by qRT-PCR. All data were normalized to GAPDH and uninfected control cells (set to 1). Bars indicate the standard error. Each experiment was performed three times; a representative experiment is shown. Note that no activation of ISG56 can be detected 8 hours after SeV and 4 hours after Reo virus infection.
(TIF)

S11 Fig Efficient MAVS cleavage, independent from its subcellular localization in HCV-infected cells. (A) A Huh7 cell line with a CRISPR/Cas-mediated knockout of MAVS was generated and transduced with lentiviral vectors encoding wt-, pex- or mitoMAVS^CR^. Cells were infected with the HCV isolate Jc1 (MOI = 1) and 48 hours later fixed and processed for immunofluorescence using antibodies specific for MAVS (red) or NS5A (green). As control non-infected (naïve) cells were processed as described above. Scale bar, 100 μm. Note that the sporadic signals obtained with MAVS^KO^ cells are background frequently localizing in-between the cells. (B) Quantitative analysis of HCV NS5A and MAVS abundance in cells expressing cleavage resistant MAVS (C508R) or the cleavage sensistive (C508) wtMAVS. For quantification at least 1,000 HCV-infected cells per condition were analyzed. Bars indicate the standard error. Note the complete absence of MAVS signal in infected cells expressing cleavage sensitive MAVS.
(TIF)

S12 Fig Correlation of mitoMAVS amounts with the degree of activation of the IFN response. (A) Huh7-MAVS^KO^ cells expressing mitoMAVS^CR^ were transfected with MAVS-targeting siRNA using concentrations specified in the top of each lane (nM). A non-targeting control siRNA served as reference (siCtrl). Protein levels of mitoMAVS^CR^ were determined by Western blot; β-actin served as loading control. (B) Abundance of mitoMAVS^CR^ as determined by immunofluorescence. Nuclei were counterstained with DAPI. Scale bar: 100 μm. (C) Cells treated as described above were infected with SeV (MOI = 5). To allow comparison of the magnitude of the IFN response, Huh7-MAVS^KO^ expressing pexMAVS^CR^ and transfected with highest amounts of control siRNA were included. RNA was extracted and mRNA amounts of ISG56 were determined by qRT-PCR. All data were normalized to GAPDH using the ΔΔct method and are expressed relative to uninfected control cells.
(TIF)

S13 Fig Limited IFN response of parental Huh7 cells used to generate MAVS^KO^ cell lines. The parental Huh7 cell line used to generate the Huh7-MAVS^KO^ cell lines, the Huh7-MAVS^KO^ cell clone itself and, as reference, the well-established Huh7-Lunet cell line [84] were treated with IFN-α (100 IU/ml) or IFN-λ1 (20 ng/ml) for 8 hours. RNA was extracted and mRNA amounts of MxA and ISG56 were determined by qRT-PCR. All data were normalized to GAPDH using the ΔΔct method and are expressed relative to uninfected control cells.
(TIF)
