# Supplementary figures and images for: Activation of Type I and III Interferon Response by Mitochondrial and Peroxisomal MAVS and Inhibition by Hepatitis C Virus
Source: PLoS Pathog. 2015 Nov 20;11(11):e1005264. doi: 10.1371/journal.ppat.1005264 (PMC4654527; doi:10.1371/journal.ppat.1005264)

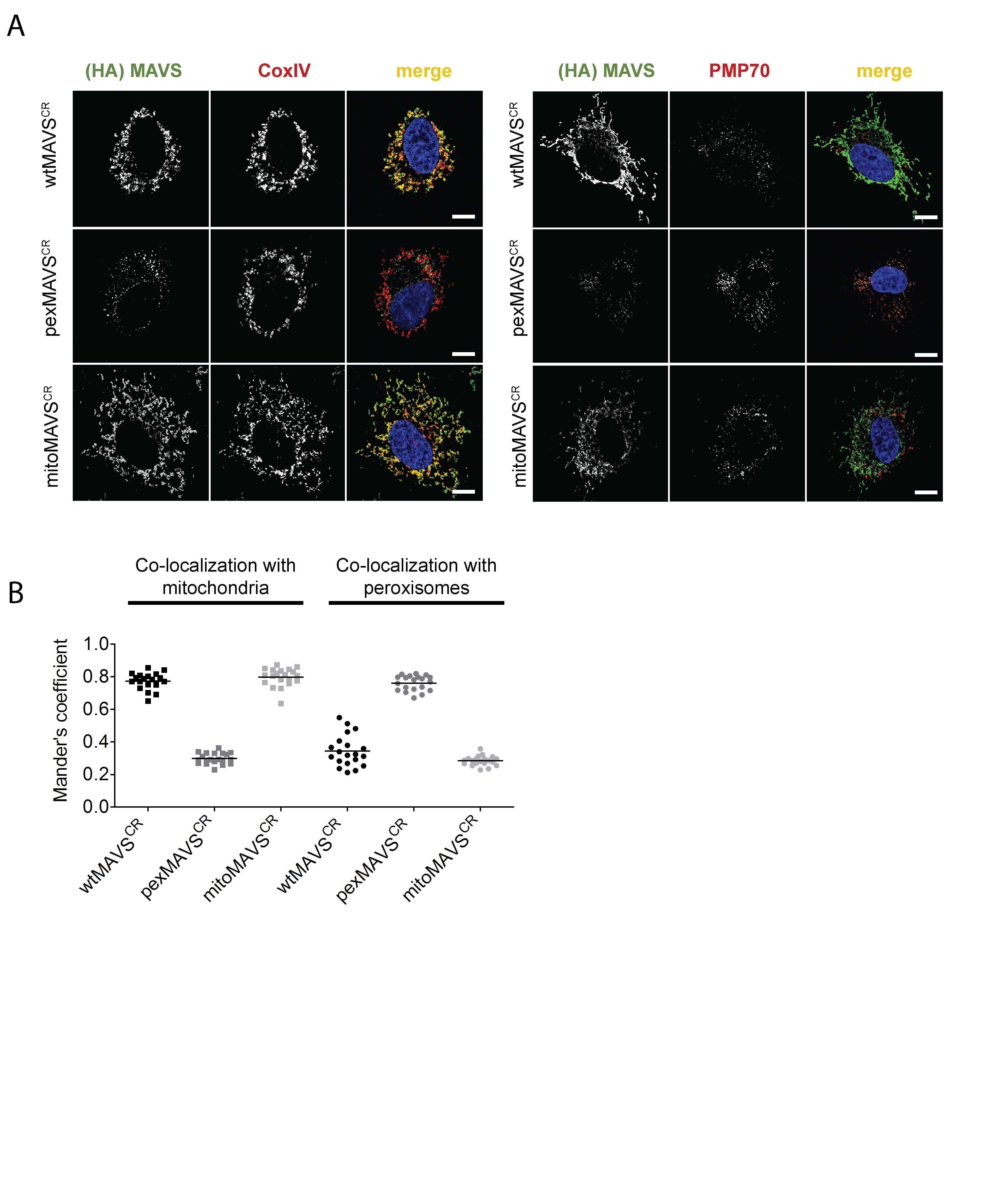

Supplement: S1 Fig — (A) A549 cells stably expressing HCV NS3/4A were transduced with lentiviral vectors encoding for wt-, pex- or mitoMAVSCR-HA (green). Cells were analyzed by immunofluorescence using antibodies specified in the top. Nuclear DNA was stained with DAPI. Scale bar, 20 μm. (B) The degree of co-localization was quantified using Mander’s overlap coefficient; each dot represents a single cell. Twenty cells were analyzed per MAVS variant. (TIF) [file ppat.1005264.s001.tif]

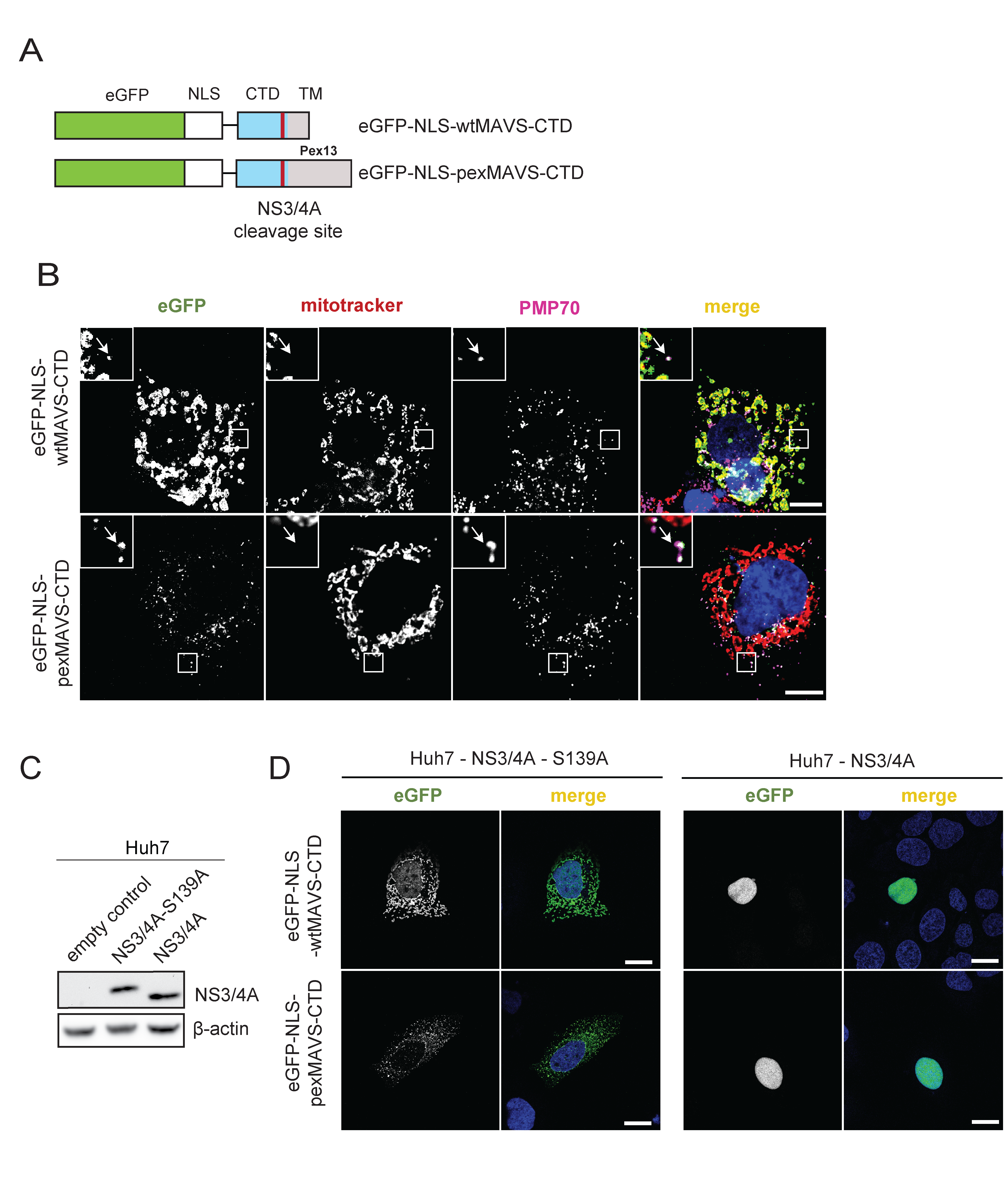

Supplement: S2 Fig — (A) Schematic representation of MAVS reporter constructs. The fusion proteins are composed of eGFP containing a nuclear localization signal (NLS), the C-terminal region (CT) of MAVS (light blue) spanning the NS3/4A protease cleavage site (red vertical line) and the wt or pex transmembrane (TM) domain for subcellular targeting. (B) Huh7 cells were transfected with eGFP-NLS-wtMAVS-CTD or eGFP-NLS-pexMAVS-CTD constructs. Co-localization of the reporter proteins with mitochondria (mitotracker, red) and peroxisomes (PMP70, purple) was determined by immunofluorescence. Scale bar, 10 μm. (C) Expression of the catalytically inactive HCV protease NS3/4A-S139A and the parental NS3/4A in stably transduced Huh7 cells was confirmed by Western blot; β-actin served as loading control. Empty control refers to cells transduced with the empty vector. (D) Huh7 cells expressing the inactive mutant (NS3/4A-S139A; left panel) or the parental protease (right panel) were transfected with expression constructs specified on the left and 24 hours later cells were fixed and analyzed by fluorescence microscopy. Nuclei were stained with DAPI. Scale bar, 20 μm. (TIF) [file ppat.1005264.s002.tif]

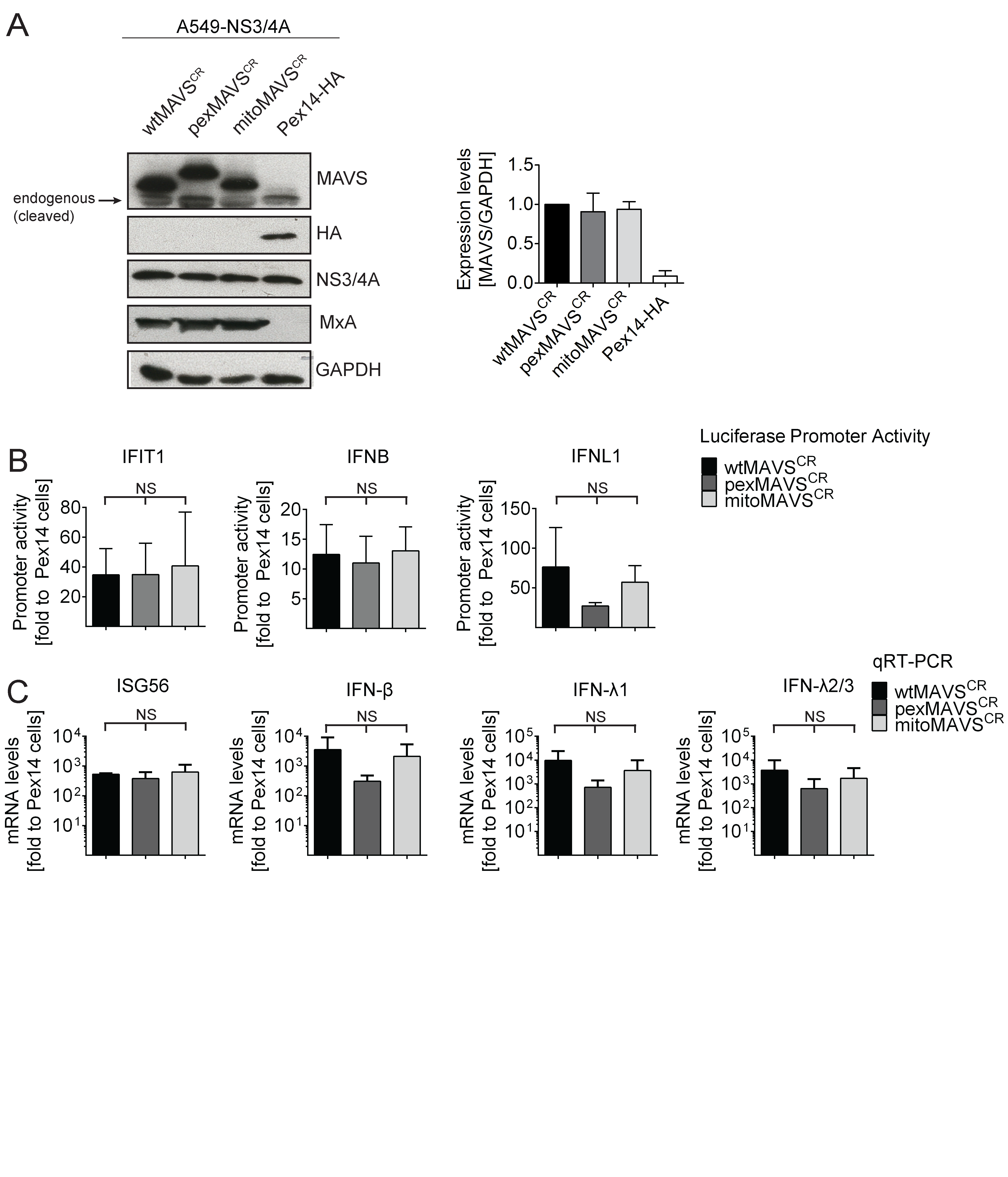

Supplement: S3 Fig — (A) A549-NS3/4A expressing cells were transduced with lentiviruses containing non-cleavable wt-, pex- or mitoMAVSCR as well as Pex14-HA that served as control. Cells were lysed 36 hours after transduction and expression of MAVSCR, endogenous cleaved MAVS and MxA were determined by Western blot using antibodies specified in the right of each panel. The relative expression levels of MAVS normalized to GAPDH and wtMAVSCR (set to 1) are shown in the right panel (mean values of three independent experiments and standard errors). (B-C) Cells were stimulated by overexpressing MAVS variants. (B) To determine activation of the IFIT1, IFNB and IFNL1 promoter (left, middle and right panel, respectively), cells were transfected with firefly luciferase reporter plasmids and a SV40-based Renilla luciferase plasmid to normalize for transfection efficiency. After 24 hours cells were lysed and luciferase activity was measured. (C) qRT-PCR analysis was performed to determine mRNA levels for the genes specified in the top of each panel. Values were normalized to GAPDH by using the ΔΔct method. Data represent the mean from four independent experiments. Bars indicate the standard error. *, P≤0.05; **, P≤0.005; ***, P≤0.0005; NS, not significant. (TIF) [file ppat.1005264.s003.tif]

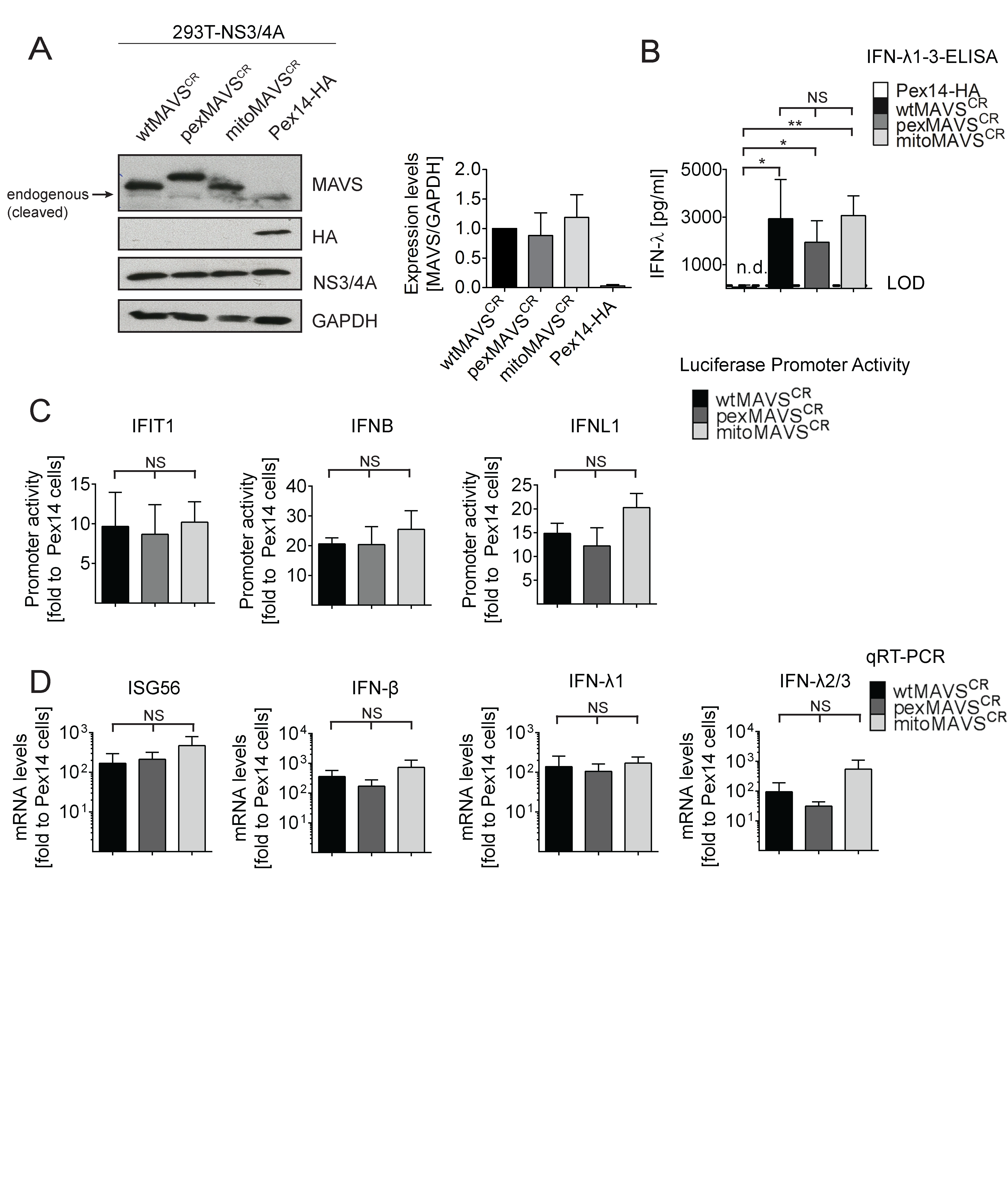

Supplement: S4 Fig — (A) 293T cells stably expressing NS3/4A were transduced with lentiviral vectors encoding for non-cleavable wt-, pex- or mitoMAVSCR as well as Pex14-HA that served as negative control. Expression of transduced genes was determined by Western blot. The quantification in the right panel displays MAVS expression levels normalized to β-actin and wtMAVSCR that was set to one. Mean values and standard error of three independent experiments are given. (B) Thirty six hours after transduction cell culture supernatants were collected to measure IFN-λ1–3 protein levels by ELISA. Dashed line represents the limit of detection (LOD). N.d., not detectable. (C) To measure promoter activation firefly luciferase reporter plasmids specified on the top of each panel were co-transfected with a SV40-based Renilla luciferase plasmid. Values were normalized to those obtained for Pex14-HA (set to 1). (D) Amounts of mRNAs specified in the top of each panel were quantified by qRT-PCR. All data were normalized to the housekeeping gene GAPDH using the ΔΔct method. Bars indicate the standard error. All experiments were performed at least three times independently. *, P≤0.05; **, P≤0.005; ***, P≤0.0005; NS, not significant. (TIF) [file ppat.1005264.s004.tif]

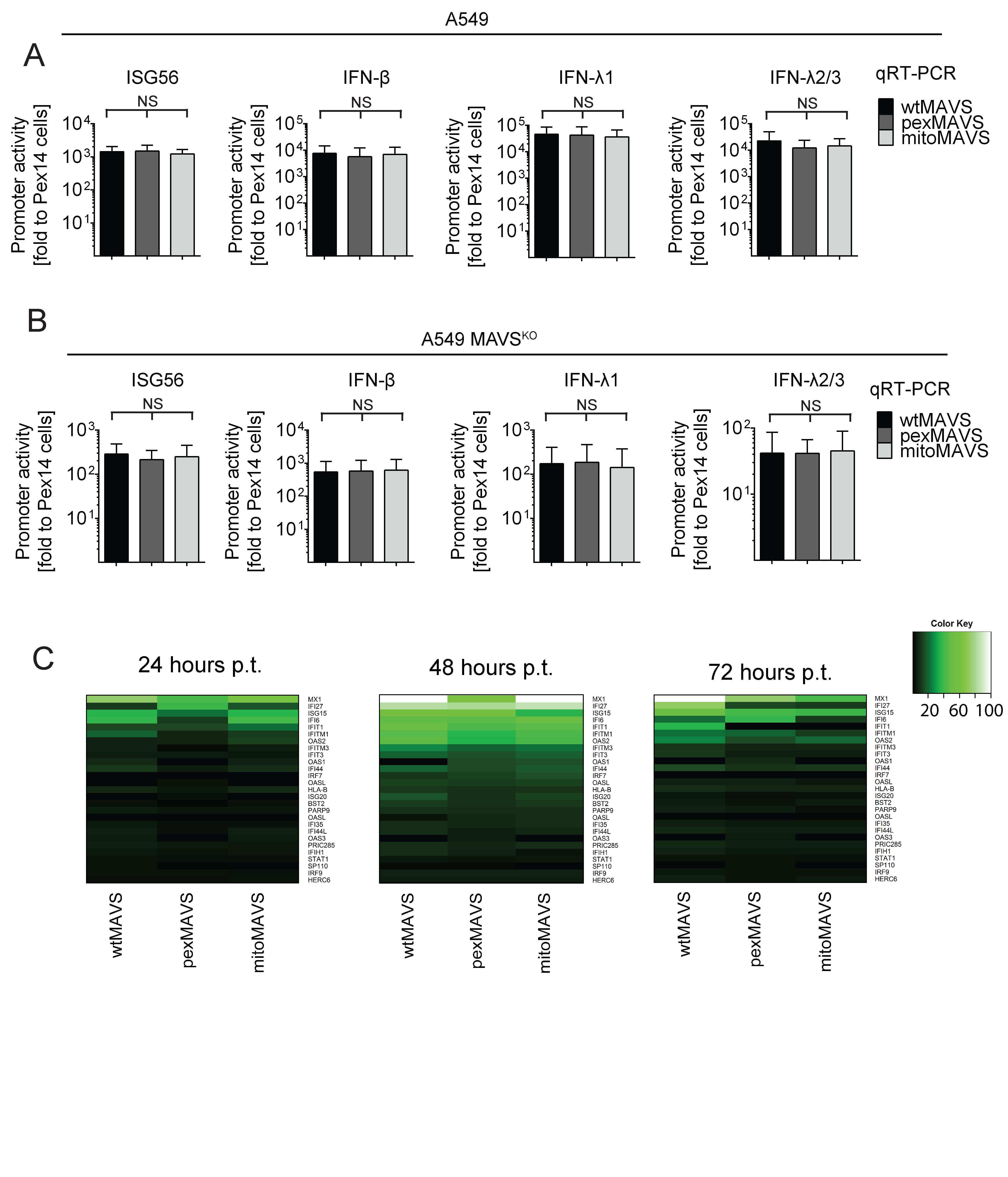

Supplement: S5 Fig — (A) A549 and (B) A549-MAVSKO cells generated by a CRISPR/Cas9 approach were transduced with lentiviral vectors encoding for wt-, pex- or mitoMAVSCR as well as Pex14-HA that served as negative control and was used for normalization. Amounts of mRNAs specified in the top of each panel were quantified by qRT-PCR. All data were normalized to the housekeeping gene GAPDH using the ΔΔct method. Bars indicate the standard error. All experiments were performed at least three times independently. NS, not significant. (C) A549-MAVSKO cells were transduced with lentiviral vectors encoding wt-, pex- or mito-MAVS and Pex14-HA as control. Total RNA was isolated 24, 48 and 72 hours post transduction (p.t.) and analyzed using whole-genome microarrays. Colors indicate the fold increase relative to Pex14-HA transduced cells. Every condition was performed in three independent experiments. (TIF) [file ppat.1005264.s005.tif]

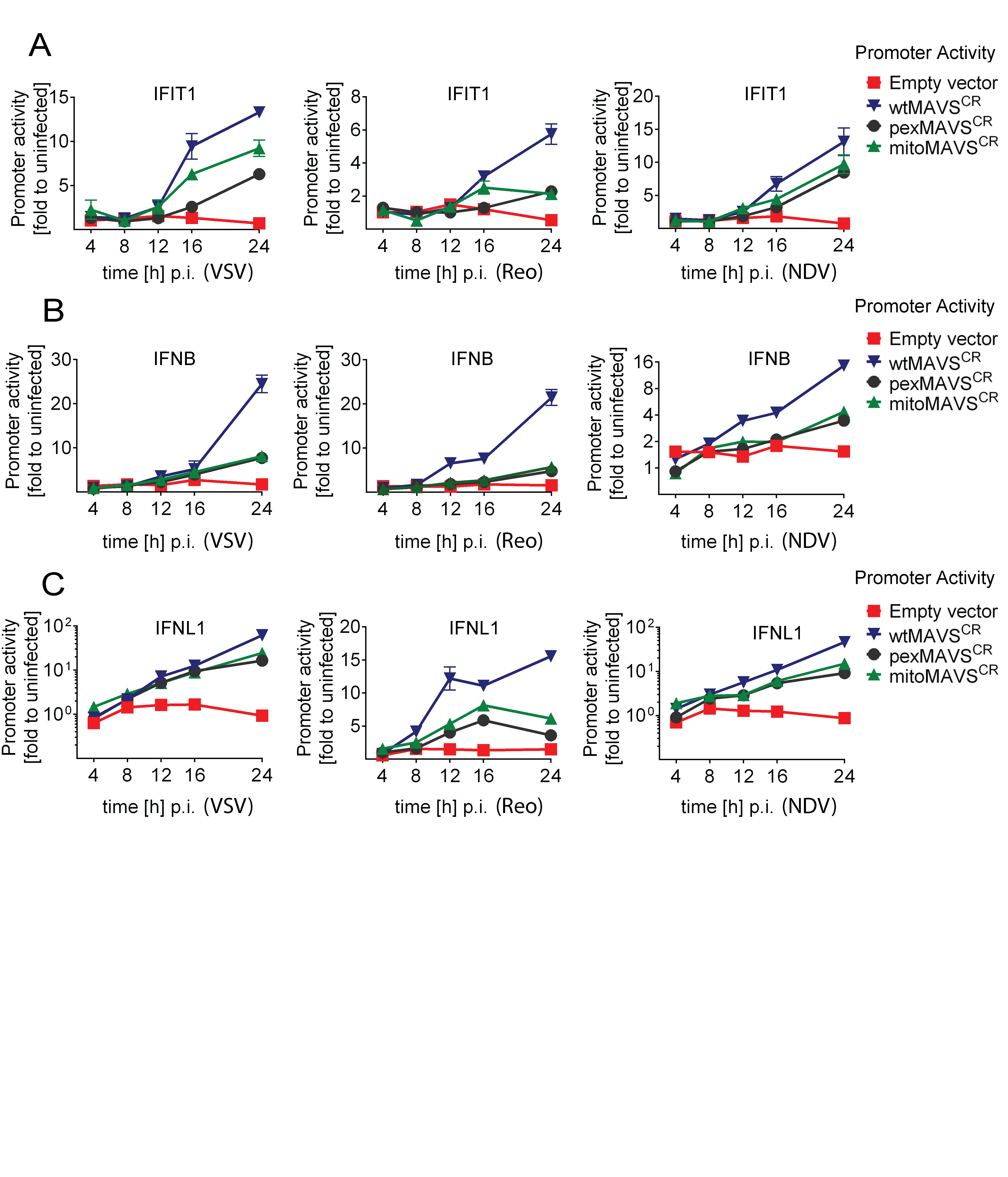

Supplement: S6 Fig — 293T cells stably expressing NS3/4A and non-cleavable wt-, pex- or mitoMAVSCR or an empty vector were infected with VSV (MOI = 3), Reo virus (MOI = 50) or NDV (MOI = 5). Twenty four hours prior to infection cells were transfected with firefly luciferase reporter plasmids specific for (A) IFIT1, (B) IFNB or (C) IFNL1. A SV40-based Renilla-luciferase plasmid was co-transfected as control to normalize for transfection efficiency. Values for each reporter were normalized to uninfected control cells that were set to one. Each panel displays a representative experiment, each conducted with technical quadruplicates. Shown are the means and standard errors (not visible in all graphs because of low variation). All experiments were conducted three times. (TIF) [file ppat.1005264.s006.tif]

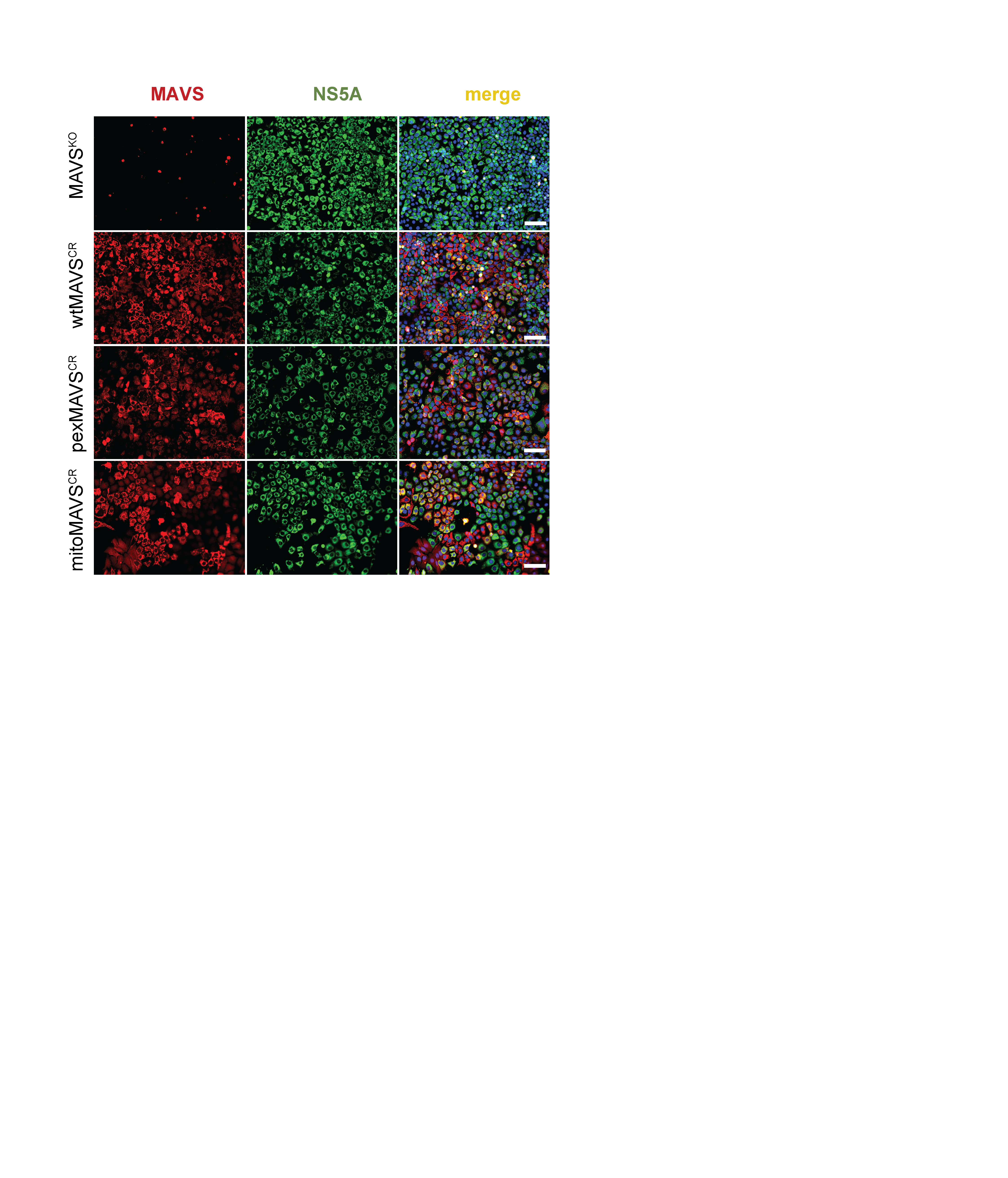

Supplement: S7 Fig — A Huh7 cell line with a CRISPR/Cas-mediated knockout of MAVS was generated and transduced with lentiviral vectors encoding cleavage resistant wt-, pex- or mitoMAVSCR. Cells were infected with the HCV isolate Jc1 (MOI = 1) and 48 hours later fixed and processed for immunofluorescence using antibodies recognizing MAVS (red) and NS5A (green). Scale bar, 100 μm. Note that the sporadic signals obtained with MAVSKO cells are background frequently localizing in-between the cells. (TIF) [file ppat.1005264.s007.tif]

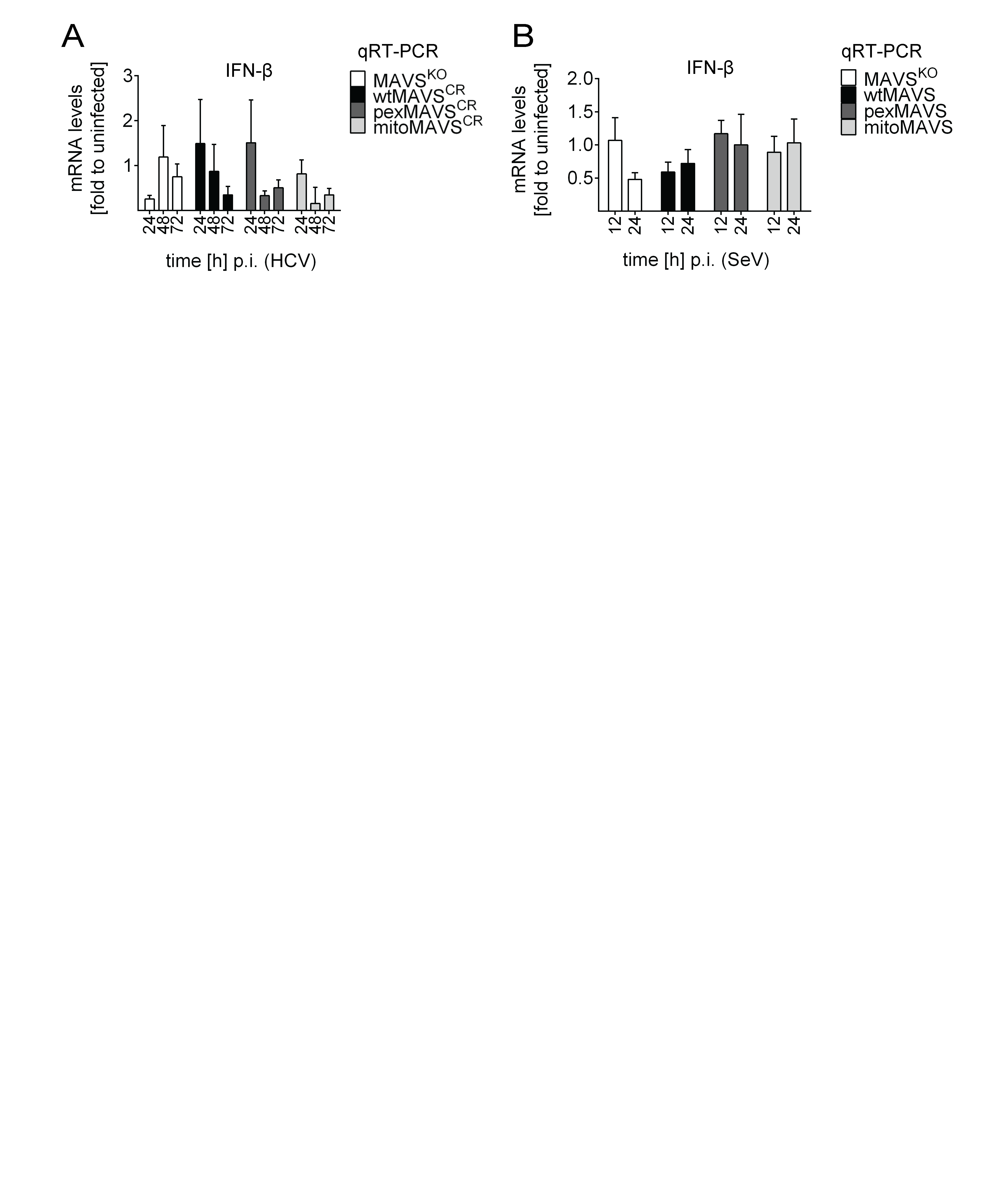

Supplement: S8 Fig — (A) Huh7 MAVSKO cell lines expressing cleavage resistant wt-, pex- and mitoMAVS were infected with the HCV isolate Jc1 (MOI = 5). Total RNA was extracted and mRNA amounts of IFN-β were determined by qRT-PCR. All data were normalized to GAPDH using the ΔΔct method and are expressed relative to uninfected control cells (set to 1). (B) Analogous to panel (A), but cells were infected with SeV (MOI = 5). For each panel, a representative experiment performed with technical triplicates is shown. Bars indicate the standard error. All experiments were conducted three times and with two additional Huh7 knockout cell clones. (TIF) [file ppat.1005264.s008.tif]

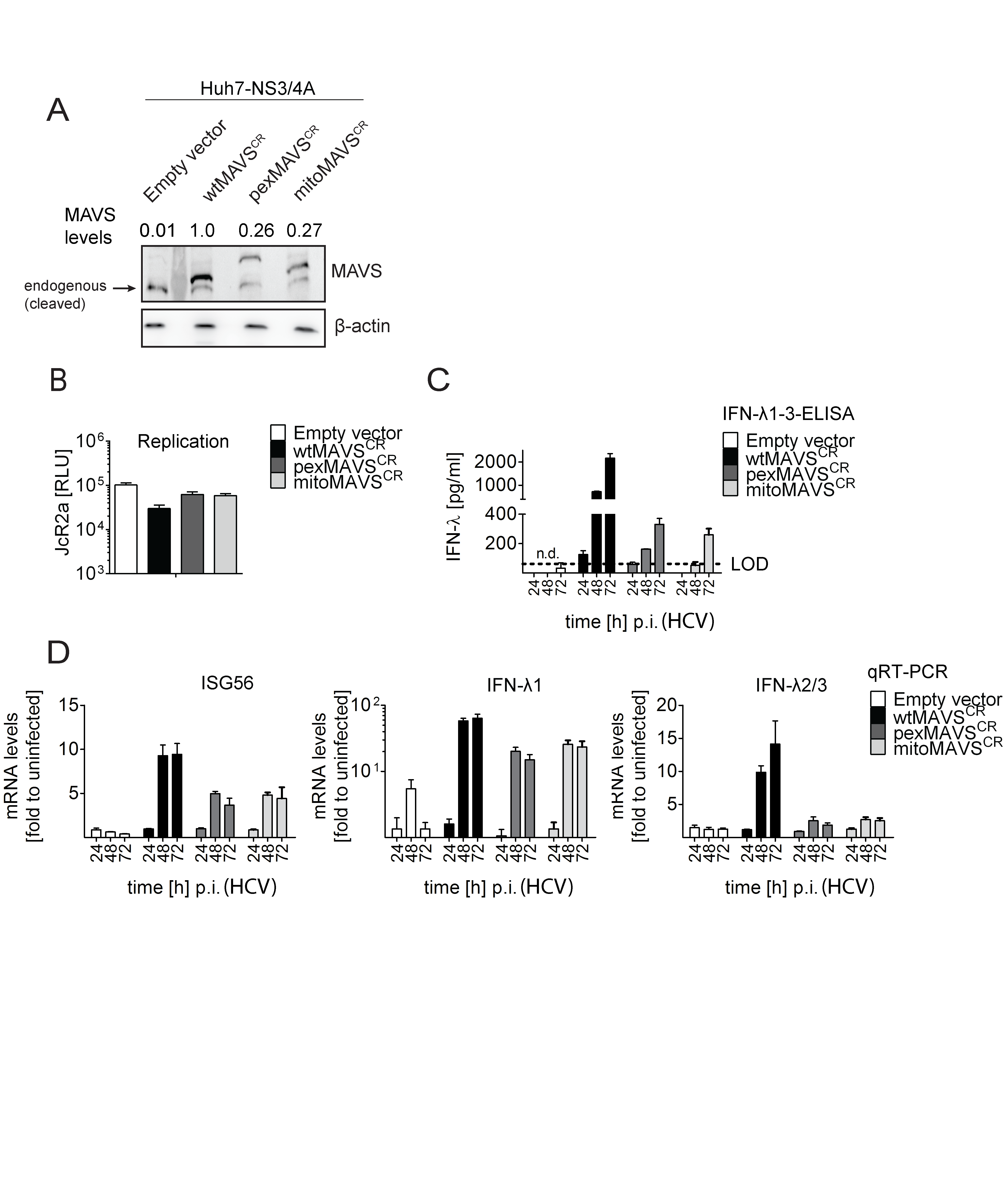

Supplement: S9 Fig — (A) Huh7 cells stably expressing NS3/4A and non-cleavable wt-, pex- or mitoMAVSCR or an empty vector were lysed and expression levels of endogenous MAVS and MAVS variants was determined by Western blot. Note the quantitative cleavage of endogenous MAVS by the HCV protease. β-actin served as loading control. Numbers above each lane refer to abundance of a given MAVS variant, normalized to β-actin and wtMAVSCR that was set to one. (B) Cells were infected with the HCV reporter virus JcR2a (MOI = 1) and Luciferase activity was measured to monitor replication 72 hours post infection. (C) Huh7-NS3/4A-MAVSCR cells were infected with the HCV isolate Jc1 (MOI = 5). Culture supernatants were harvested at time points specified in the bottom of the graph and amount of IFN-λ1–3 was determined by ELISA. The dashed line represents the limit of detection (LOD). N.d., not detectable. (D) HCV-infected cells were harvested at given time points, total RNA was extracted and mRNA levels of ISG56, IFN-λ1 and IFN-λ2/3 were determined by qRT-PCR. All data were normalized to GAPDH using the ΔΔct method and to uninfected control cells (set to 1). Shown are the means of a representative experiment, each time point measured in triplicates. Bars indicate the standard error. All experiments were done three times. (TIF) [file ppat.1005264.s009.tif]

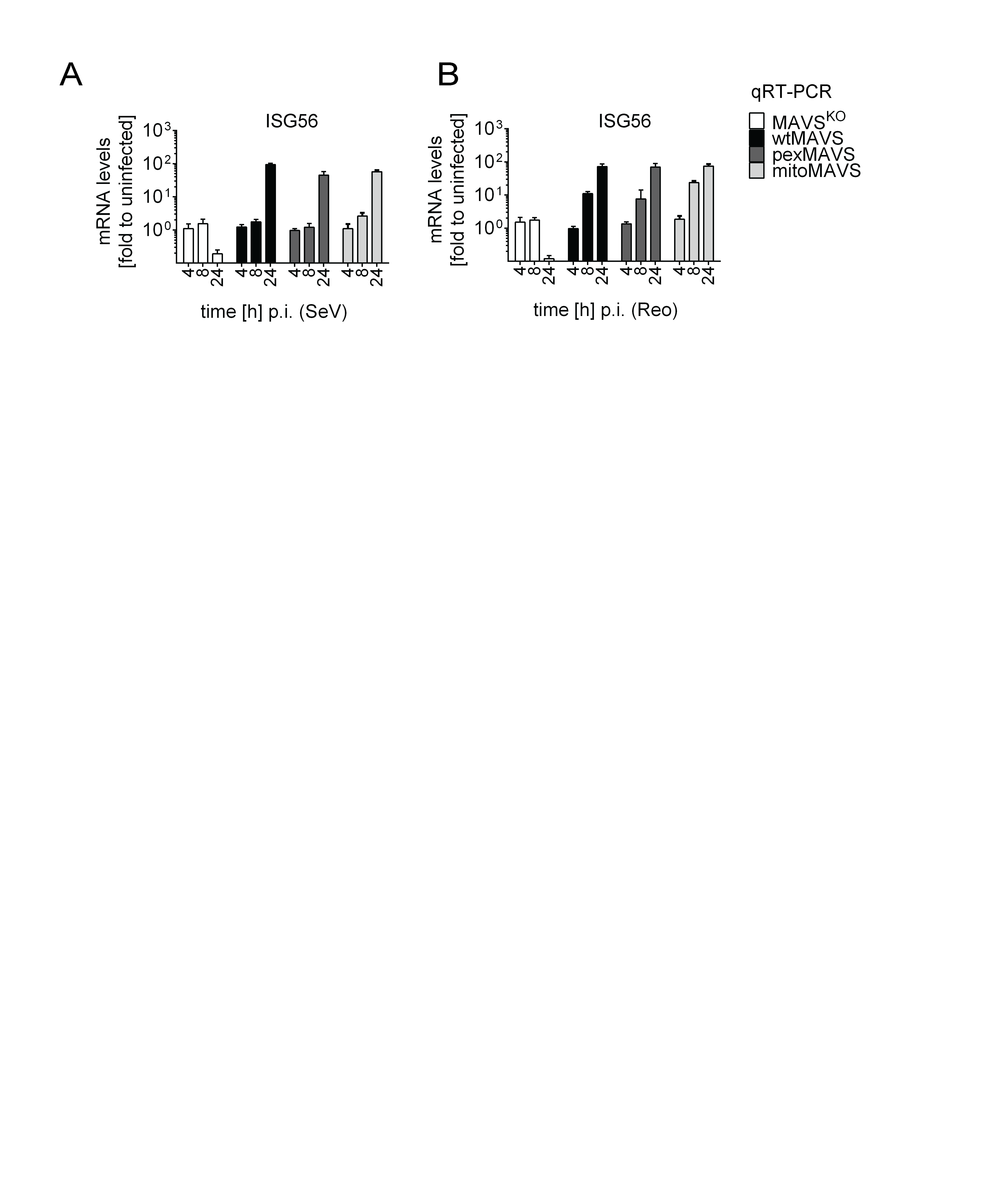

Supplement: S10 Fig — Huh7-MAVSKO cells were rescued with cleavable wt-, pex- or mitoMAVS or cleavage-resistant wtMAVSCR. (A) Cells were infected with SeV (MOI = 5) or (B) Reo virus (MOI = 50) and harvested at different time points after infection that are specified in the bottom of each panel. Total RNA was isolated and amounts of ISG56 mRNA were determined by qRT-PCR. All data were normalized to GAPDH and uninfected control cells (set to 1). Bars indicate the standard error. Each experiment was performed three times; a representative experiment is shown. Note that no activation of ISG56 can be detected 8 hours after SeV and 4 hours after Reo virus infection. (TIF) [file ppat.1005264.s010.tif]

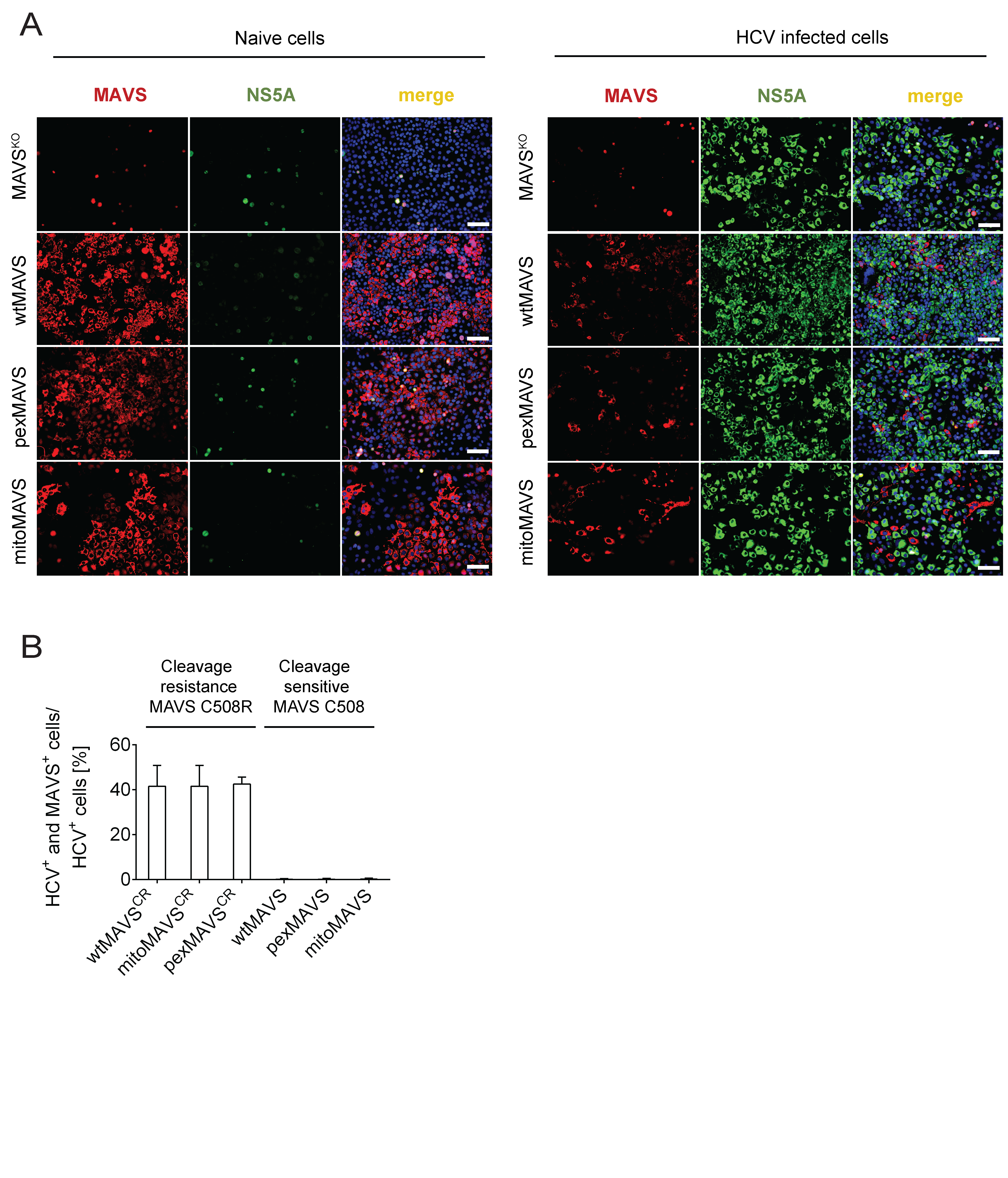

Supplement: S11 Fig — (A) A Huh7 cell line with a CRISPR/Cas-mediated knockout of MAVS was generated and transduced with lentiviral vectors encoding wt-, pex- or mitoMAVSCR. Cells were infected with the HCV isolate Jc1 (MOI = 1) and 48 hours later fixed and processed for immunofluorescence using antibodies specific for MAVS (red) or NS5A (green). As control non-infected (naïve) cells were processed as described above. Scale bar, 100 μm. Note that the sporadic signals obtained with MAVSKO cells are background frequently localizing in-between the cells. (B) Quantitative analysis of HCV NS5A and MAVS abundance in cells expressing cleavage resistant MAVS (C508R) or the cleavage sensistive (C508) wtMAVS. For quantification at least 1,000 HCV-infected cells per condition were analyzed. Bars indicate the standard error. Note the complete absence of MAVS signal in infected cells expressing cleavage sensitive MAVS. (TIF) [file ppat.1005264.s011.tif]

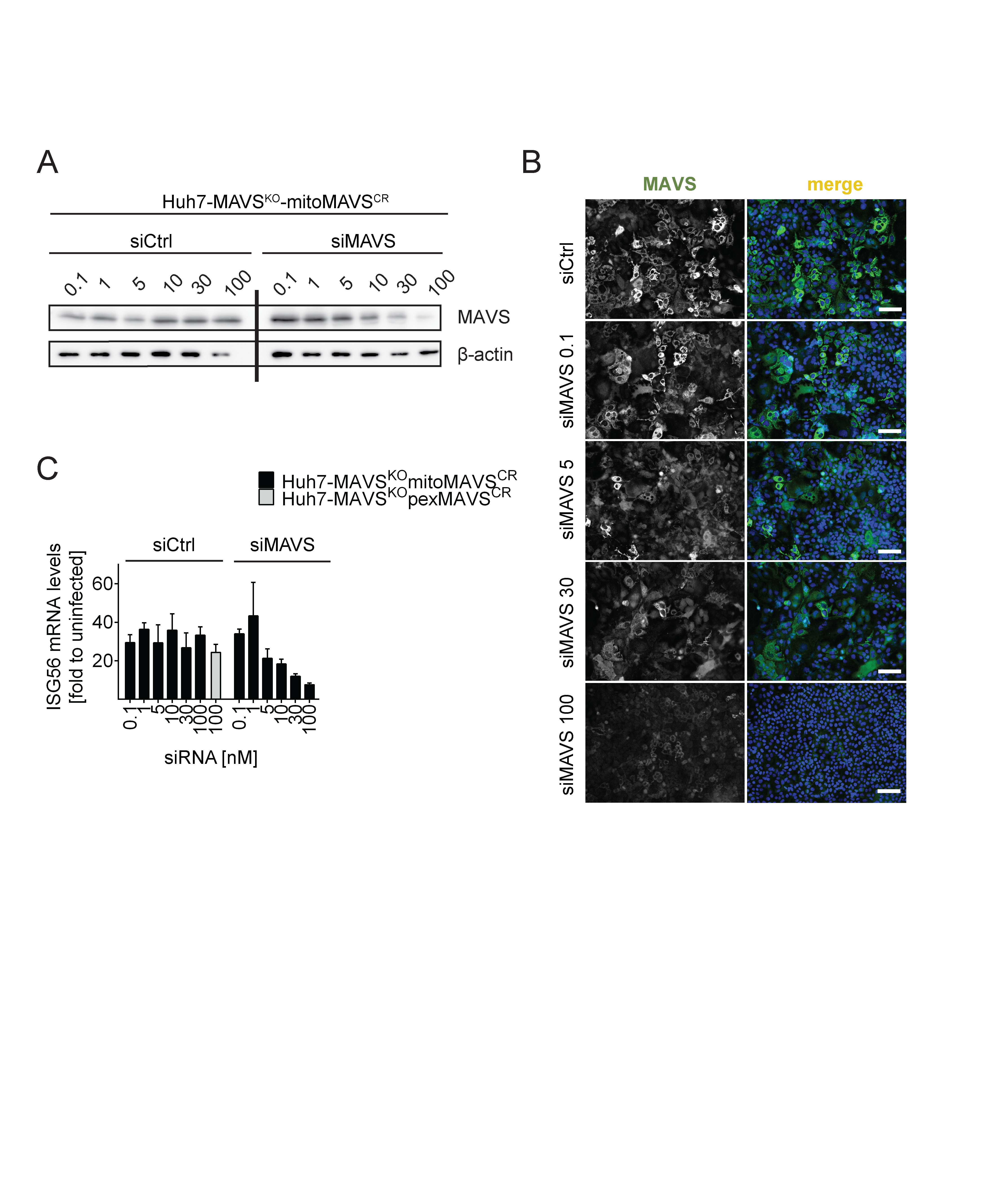

Supplement: S12 Fig — (A) Huh7-MAVSKO cells expressing mitoMAVSCR were transfected with MAVS-targeting siRNA using concentrations specified in the top of each lane (nM). A non-targeting control siRNA served as reference (siCtrl). Protein levels of mitoMAVSCR were determined by Western blot; β-actin served as loading control. (B) Abundance of mitoMAVSCR as determined by immunofluorescence. Nuclei were counterstained with DAPI. Scale bar: 100 μm. (C) Cells treated as described above were infected with SeV (MOI = 5). To allow comparison of the magnitude of the IFN response, Huh7-MAVSKO expressing pexMAVSCR and transfected with highest amounts of control siRNA were included. RNA was extracted and mRNA amounts of ISG56 were determined by qRT-PCR. All data were normalized to GAPDH using the ΔΔct method and are expressed relative to uninfected control cells. (TIF) [file ppat.1005264.s012.tif]

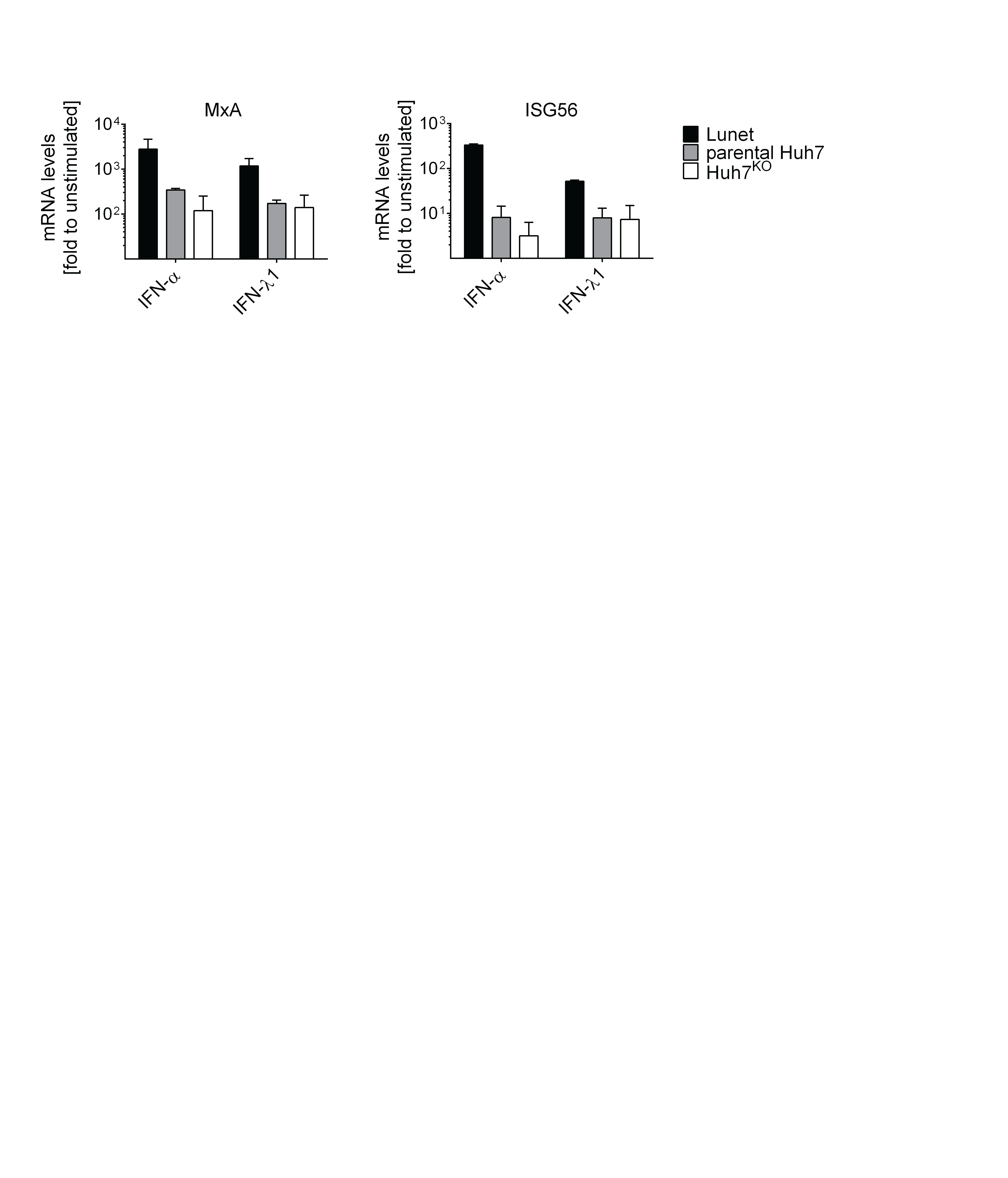

Supplement: S13 Fig — The parental Huh7 cell line used to generate the Huh7-MAVSKO cell lines, the Huh7-MAVSKO cell clone itself and, as reference, the well-established Huh7-Lunet cell line [84] were treated with IFN-α (100 IU/ml) or IFN-λ1 (20 ng/ml) for 8 hours. RNA was extracted and mRNA amounts of MxA and ISG56 were determined by qRT-PCR. All data were normalized to GAPDH using the ΔΔct method and are expressed relative to uninfected control cells. (TIF) [file ppat.1005264.s013.tif]
